# Aspects of gorgonopsian paleobiology and evolution: insights from the basicranium, occiput, osseous labyrinth, vasculature, and neuroanatomy

**DOI:** 10.7717/peerj.3119

**Published:** 2017-04-11

**Authors:** Ricardo Araújo, Vincent Fernandez, Michael J. Polcyn, Jörg Fröbisch, Rui M.S. Martins

**Affiliations:** 1 Instituto Superior Técnico, Instituto de Plasmas e Fusão Nuclear, Universidade de Lisboa, Lisboa, Portugal; 2 Museum für Naturkunde, Leibniz-Institut für Evolutions—und Biodiversitätsforschung, Berlin, Germany; 3 Huffington Department of Earth Sciences, Southern Methodist Univesity, Dallas, TX, United States of America; 4 GEAL—Museu da Lourinhã, Lourinhã, Portugal; 5 Institut des Sciences de l’Evolution, Université de Montpellier 2, Montpellier, France; 6 European Synchrotron Radiation Facility, Grenoble, France; 7 Institut für Biologie, Humboldt-Universität zu Berlin, Berlin, Germany; 8 CENIMAT/I3N, Universidade Nova de Lisboa, Monte de Caparica, Portugal

**Keywords:** Braincase, Mammals, Modularity, Vestibular organ, Synchrotron, Gorgonopsian, Homology, Brain, Vasculature, Therapsid

## Abstract

Synapsida, the clade including therapsids and thus also mammals, is one of the two major branches of amniotes. Organismal design, with modularity as a concept, offers insights into the evolution of therapsids, a group that experienced profound anatomical transformations throughout the past 270 Ma, eventually leading to the evolution of the mammalian bauplan. However, the anatomy of some therapsid groups remains obscure. Gorgonopsian braincase anatomy is poorly known and many anatomical aspects of the brain, cranial nerves, vasculature, and osseous labyrinth, remain unclear. We analyzed two gorgonopsian specimens, GPIT/RE/7124 and GPIT/RE/7119, using propagation phase contrast synchrotron micro-computed tomography. The lack of fusion between many basicranial and occipital bones in GPIT/RE/7124, which is an immature specimen, allowed us to reconstruct its anatomy and ontogenetic sequence, in comparison with the mature GPIT/RE/7119, in great detail. We explored the braincase and rendered various skull cavities. Notably, we found that there is a separate ossification between what was previously referred to as the “parasphenoid” and the basioccipital. We reinterpreted this element as a posterior ossification of the basisphenoid: the basipostsphenoid. Moreover, we show that the previously called “parasphenoid” is in fact the co-ossification of the dermal parasphenoid and the endochondral basipresphenoid. In line with previous descriptions, the anatomy of the osseous labyrinth is rendered in detail, revealing a unique discoid morphology of the horizontal semicircular canal, rather than toroidal, probably due to architectural constraints of the ossification of the opisthotic and supraoccipital. In addition, the orientation of the horizontal semicircular canal suggests that gorgonopsians had an anteriorly tilted alert head posture. The morphology of the brain endocast is in accordance with the more reptilian endocast shape of other non-mammaliaform neotherapsids.

## Introduction

Radical transformations in the synapsid skull arrangement led to the unique mammalian cranial design; however, the inner skull anatomy of some therapsid groups such as the gorgonopsians remains mostly unknown. Moreover, although many sensory systems leave fossil evidence in the braincase, the gorgonopsian braincase remains an obscure element of the pre-mammalian evolutionary record. This scarcity in detailed descriptions of the gorgonopsian braincase can be partly attributed to technological constraints. Indeed, while it is believed that gorgonopsians have a high degree of cranial homomorphism (Sigogneau-Russell, 1989; Kammerer, 2016), our knowledge of the braincase is largely based on external morphology and on destructive serial grinding. Several braincase elements are rarely exposed (e.g., prootic, epipterygoid; Kammerer, 2016) and even in the acid-prepared skulls described by Kemp (1969) the descriptions are terse. The ventral surface of the palate is the only basicranial aspect that has been thoroughly studied (Sigogneau-Russell, 1989; Kammerer, 2014). However, many features related to the neuroanatomy and sensory systems are on the dorsal surface of the basicranium and anterior surface of the occiput. Olson (1938), Kemp (1969) and Sigogneau (1970) count among the few studies that provided significant insights into the basicranium and occiput. Nevertheless, many uncertainties remain as these braincase reconstructions mostly resulted from analyses of serial sectioning or specimens broken along a single plan. The gorgonopsian braincase is complex, particularly in older individuals where extensive co-ossification and fusion has taken place, thus making rendering interpretations rather challenging.

In recent years, non-destructive imaging of rare and fragile fossil specimens has greatly benefited paleontological studies by uncovering previously inaccessible anatomy. Here, we provide a detailed description of the gorgonopsian braincase by using propagation phase-contrast synchrotron radiation-based micro-computed tomography. We selected two specimens for analysis: GPIT/RE/7124 and GPIT/RE/7119. GPIT/RE/7124, previously attributed to *Aloposaurus gracilis*, was selected because it shows several osteologically immature features including a clear separation of the basicranial elements, which are typically co-ossified in osteologically mature specimens such as GPIT/RE/7119. The braincase of GPIT/RE/7124 has never been described in detail, with the exception of the posterior view of the occiput and the ventral view of the palate (von Huene, 1937), and a more recent re-analysis of the specimen (Sigogneau-Russell, 1989). GPIT/RE/7119 is a Tanzanian specimen that was initially described as *Dixeya nasuta* by von Huene (1950), and later reclassified as *Arctognathus*? *nasuta* by Sigogneau-Russell (1989). Gebauer (2007) maintained the ascription to this genus, however, Kammerer (2015) points several differences in GPIT/RE/7119 relative to the holotype of *Arctognathus*. Thus, the taxonomic content of the genus needs to be revised. We segmented all the individual bones of the occiput and braincase of GPIT/RE/7124 and GPIT/RE/7119 where it was possible to separate them. For GPIT/RE/7124, we also segmented the voids bounded by the basicranial bones (i.e., brain endocast, osseous labyrinth, cranial nerves and vasculature). Our results offer the first detailed insights into the gorgonopsian braincase.

## Materials and Methods

### Materials

**Figure 1:**
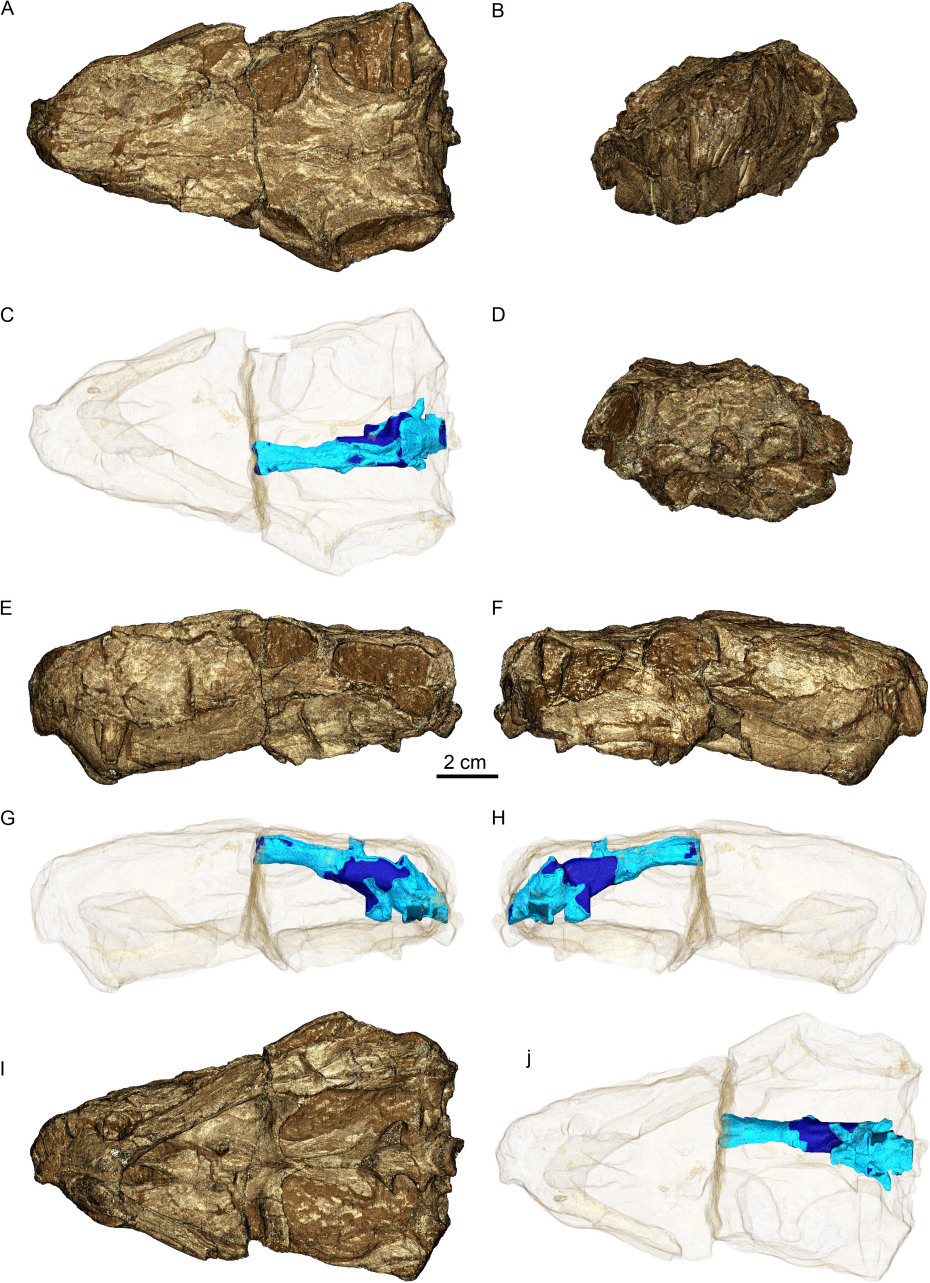
Three-dimensional rendering of GPIT/RE/7124 skull in dorsal (A), anterior (B), posterior (D), lateral left (E) and right (F) and ventral (I) views. Semi-transparent rendering of the skull with endocast (blue) in dorsal (C), lateral left (G) and right (H) and ventral (J) views. The light blue color of the endocast indicates where the segmentation was surrounded by bones unlike the dark blue parts.

GPIT/RE/7124 (Fig. 1) was collected at Heuning Nest Krantz (also spelled: Heuningneskrans or Honingnest Krantz) in the district Graaff Reinet, South Africa (von Huene, 1937), from strata considered to belong to the *Cistecephalus* Assemblage Zone (Kitching, 1977; van der Walt et al., 2010), Wuchiapingian in age (Cohen et al., 2013), and from about 255–256 Ma (Rubidge et al., 2013). von Huene (1937) ascribed this specimen to *Aloposaurus gracilis.* Unfortunately, information on the collector or the exact stratigraphic level is not provided in the original description of the specimen (von Huene, 1937). Von Huene did not collect the specimen himself, but was able to obtain it from South Africa. It was prepared further possibly in Tübingen. The braincase is not explicitly described, there is a detailed description about the posterior view of the occiput and the ventral view of the palate (von Huene, 1937), and later Gebauer (2007) redescribed the external anatomy of the specimen. Sigogneau (1970) reconsidered von Huene’s systematic placement and allocated the specimen to Gorgonopsia incertae sedis and later to *Aelurosaurus* (Sigogneau-Russell, 1989). Thereafter, Gebauer (2007) recently revised the specimen and ascribed it to *Aelurosaurus wilmanae* on the basis of the following characters: a relatively broad snout, prefrontal large but short anteriorly, broad intertemporal space, transverse apophyses without teeth, occiput less inclined than in the other species.

GPIT/RE/7119 was found from a layer more than 40 m above the lower boundary of the Tanzanian equivalent of the *Cistecephalus* zone (Angielczyk et al., 2014) near the Kingori Mountain (von Huene, 1950). von Huene (1950) describes the anatomy and relationships of the specimen attributing it to the species *Dixeya nasuta*, later revised by Sigogneau (1970) and Gebauer (2007) as *Arctognathus*? *nasuta*, however this taxonomic placement is currently under revision (Kammerer, 2015).

### Propagation phase contrast synchrotron micro-computed tomography

Both skulls GPIT/RE/7124 and GPIT/RE/7119 were scanned at the ID17 beamline of the European Synchrotron Radiation Facility (ESRF, Grenoble, France; proposal HG-23) using Propagation Phase Contrast Synchrotron Radiation-based micro-Computed Tomography. The setup consisted of a FReLoN-2k camera, a 0.3× magnification set of lenses, a scintillating fiber optic, a monochromatic X-ray beam of 100 keV and 150 keV respectively (bent double-Laue crystals) and a sample-detector distance of 10.9 m to perform Propagation Phase Contrast Synchrotron micro Computed Tomography (PPC-SR*μ*CT). Tomographies were computed based on 4,998 projections of 0.1 s for GPIT/RE/7124 and 0.2 s for GPIT/RE/7119, over 360 degrees resulting in data with an isotropic voxel size of 46.57 µm and 45.71 µm respectively. Additionally, the center of rotation was shifted to the size of the image (∼45 mm and ∼85 mm respectively) to increase the horizontal field of view in the reconstructed data (i.e., half acquisition protocol).

The tomographic reconstruction was performed using the single distance phase retrieval approach of the software PyHST2 (Paganin et al., 2002; Mirone et al., 2014). The *δ*/ß value was set to 1,000 based on trial reconstructions (range tested 500–2,000) as it was offering the best contrast for segmentation while not blurring the images. The resulting 32 bits data were converted to a stack of 16 bits tiff using 0.2 % saturation min and max values from the 3D histogram generated by PyHST2.

For GPIT/RE/7124, we performed two additional steps: first as the fossil contains large dense minerals (most likely metallic), it was not possible to adjust the contrast properly to differentiate bones from matrix without causing problematic saturation of the image. To limit this issue, we applied a high-pass filter on the dense structures, segmented using a threshold, using a 2D median with a window size of 3 pixels, preserving only edges of the dense material while decreasing their mean grey values. Secondly, as the flat field correction was not able to completely correct the vertical intensity gradient of the synchrotron beam, we applied a second 2D median filter with a window size of 250 pixels to normalize the mean grey values of each slice.

Segmentation of GPIT/RE/ 7124 was performed first on Amira 5.3 (FEI Visualization Sciences Group, Mérignac, France) on a 2 × 2 × 2 binned version of the volume to facilitate handling of the data set. We performed manual segmentation with masking and we mostly used tools like “brush” and “magic wand”. Measurements were taken using the 3D measurement tool in the actual segmentation. Secondly, the created surfaces were imported into the original, non-binned volume using VGStudio MAX 3.0 (Volume Graphics, Heidelberg, Germany). Region of interest (ROI) of bones were refined using region growing tool, bounded to the previous segmentation made on the binning version. Concerning the endocast, the ROI was dilated in 3D by 9 voxels, then smoothed with a strength of 50 pixels to ensure it would overlap surrounding bones when present and remove linear pattern from manual segmentation. We then removed the overlapping part by subtracting surrounding bone ROIs to the endocast ROI. To clearly identify parts of the endocast bounded by bones, we merged the ROIs of all bones into a single one, dilated it by 3 voxels and created a new ROI from its intersection with the endocast ROI. Then, on the final rendering of the endocast, by showing the full endocast and intersection at the same time, part truly defined by surrounding bones are clearly shown, as well as part resulting from interpolation.

Before rendering we performed a 3D median filter with a window size of 3 voxels to decrease the noise at the surface of the bones. Finally we used volume rendering with the Phong algorithm, an oversampling of 5 and a density of 2 to generate images.

Virtual cross sections of GPIT/RE/7124 were generated on VGStudio MAX 3.0, using the thick slab mode, showing the average of 3 slices to decrease overall noise on the images.

### Anatomical description of the braincase

**Figure 2:**
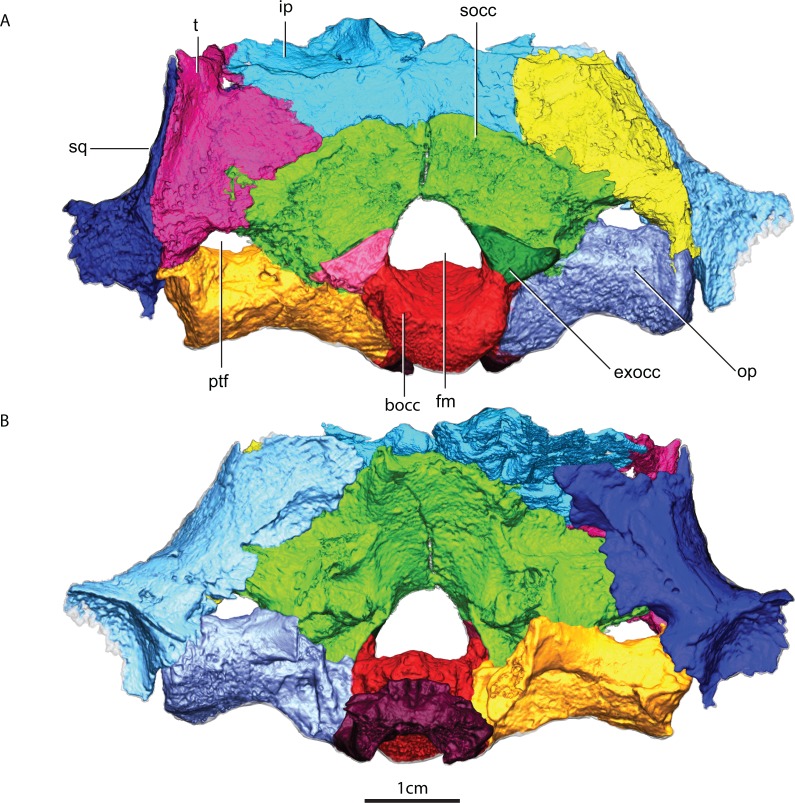
Occiput in posterior (A) and anterior views (B). Abbreviations: bocc, basioccipital; exocc, exoccipital; fm, foramen magnum; ip, interparietal; op, opisthotic; ptf, posttemporal fenestra; socc, supraoccipital; sq, squamosal; t, tabular.

**Figure 3:**
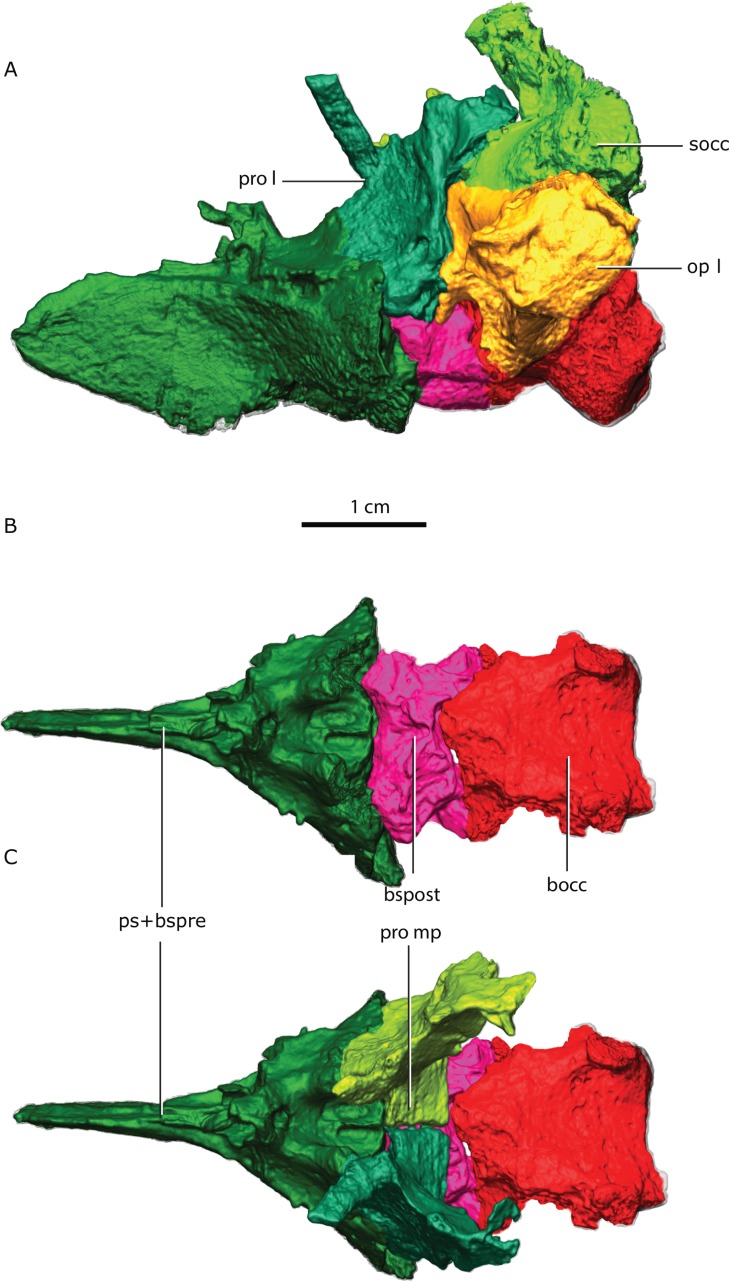
Topological arrangement of the basicranial elements in lateral (A), dorsal (B, C) views, with the parasphenoid-basipresphenoid complex anteriorly, then the basipostsphenoid overlaid by the medial process of the prootics (C), which are posteriorly bounded by the basioccipital. Abbreviations: bocc, basioccipital; bspost, basipostsphenoid; op l, left opisthotic; pro l, left prootic; pro mp, prootic medial process; ps+bspre, parasphenoid + basipresphenoid; socc, supraoccipital.

**Figure 4:**
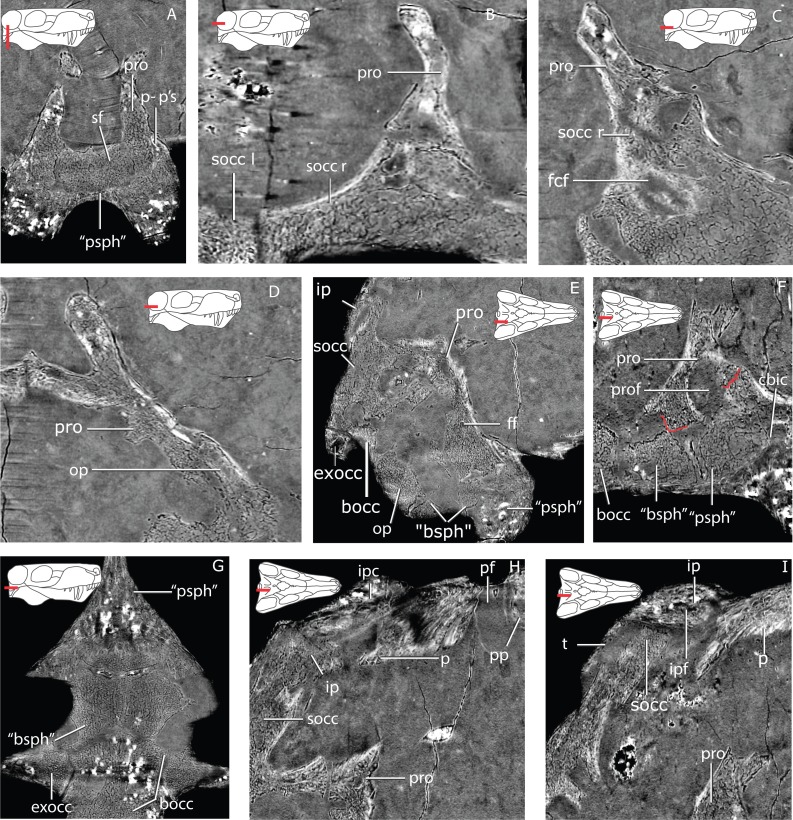
Tomographic slices for GPIT/RE7124. (A) the “parasphenoid”-prootic suture (p-p’s) and sellar floor (sf) in a coronal section; (B) the suture between the left and right supraoccipital (socc l and socc r), and the right supraoccipital and prootic (pro) in a horizontal section; (C) a more ventral view of the prootic-supraoccipital and the flocular complex fossa (fcf) in a horizontal section; (D) the prootic-opisthotic (op) suture in a horizontal section; (E) a sagittal section of the braincase showing the facial foramen (ff); (F) a more medial sagittal section showing the prootic fossa (prof) and the cerebral branch of the internal carotid (cbic) on the “parasphenoid” (“psph”); (G) horizontal section showing the sutures between the “parasphenoid”, “basisphenoid” (“bsph”), basioccipital (bocc) and exoccipital (exocc); (H) median sagittal view of the occiput showing the relationship of the tabular (t), supraoccipital, parietal (p) and interparietal (ip), notice the interparietal foramen (ipf); (I) slightly more laterally offset sagittal section showing the interparietal canal (ipc) and the relationship of the supraoccipital, interparietal and parietal. Additional abbreviations: pf, parietal foramen, pp, preparietal.

Despite some plastic deformation previously described by Sigogneau (1970), the braincase and occiput region of GPIT/RE/7124 is moderately well preserved (Figs. 2–4). GPIT/RE/7124 is somewhat dorsoventrally compressed, there is minor displacement of posterior occipital elements, and there are several fractures in the skull roof and occiput. Numerous metallic inclusions pervade through the specimen, but due to their small dimensions they did not affect segmentation. Due to fusion and fracturing, the sutures between the interparietal and tabulars were the most difficult to discern, therefore the actual morphology of these bones is here assumed to be tentative. Relevant structures, particularly those with ontogenetic importance, of the GPIT/RE/7119 are examined on the discussion, thus the following description is solely focused on the more complete GPIT/RE/7124 braincase. The terms basipresphenoid and basipostsphenoid derive from the developmental literature, where they are regarded as subdivisions of the basisphenoid (Couly, Coltey & Le Douarin, 1993).

### Prootic

The right and left prootic are exquisitely preserved, providing new anatomical information. Only the anterior part of the right pila antotica (but see Kemp’s 1969 opinion on the origin of this structure) did not preserve and the left anterodorsal process (of Kemp, 1969, *taenia marginalis* of Sigogneau-Russell, 1989) is incompletely preserved in the left prootic.

The basal region contacts the parasphenoid-basipresphenoid anteriorly, the basipostsphenoid ventrally, the supraoccipital posterodorsally, the opisthotic posteroventrally, and the contralateral prootic medially. Although the anterior portion of the basioccipital extends far anteriorly, it does not contact the prootic (Figs. 2 and 3). In the sellar region, posterior to the excavation on the basipresphenoid for the sella turcica, the prootic is conspicuously excavated laterally, forming the prootic embayment.

**Figure 5:**
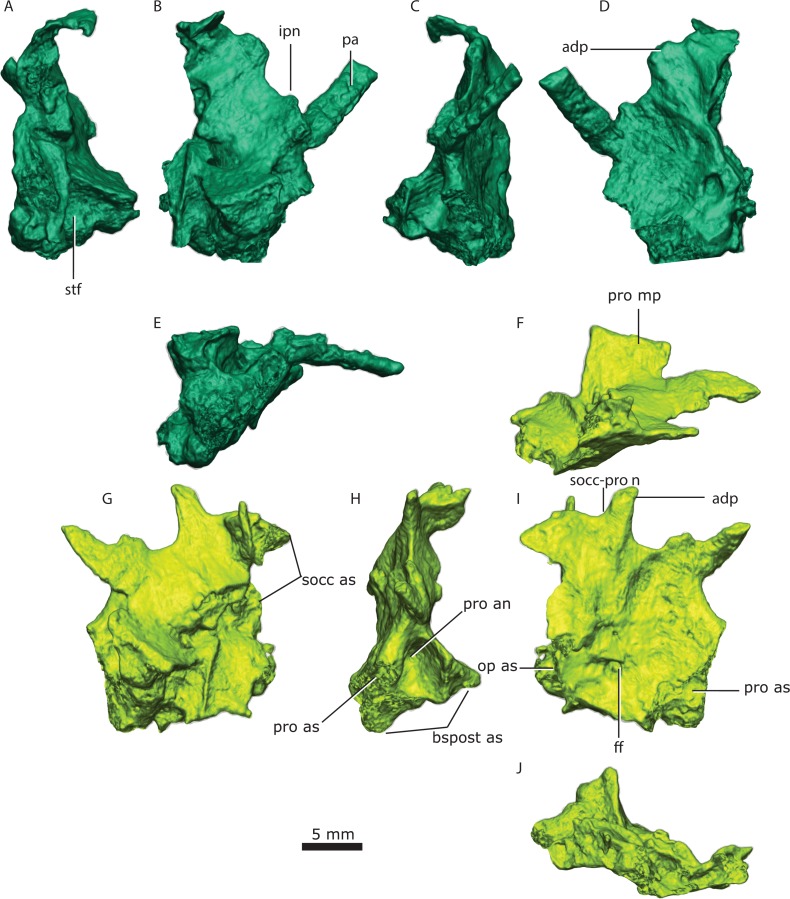
Left prootic in posterior (A), medial (B), anterior (C), lateral (D) and ventral (E) views. Right prootic in dorsal (F), medial (G), anterior (H), lateral (I) and ventral (J) views. Abbreviations: adp, anterodorsal process; bspost as, basipostsphenoid articular surface; ff, facial foramen; ipn, interprocess notch; op as, opisthotic articular surface; pa, pila antotica; pro an, anterior prootic notch; pro as, prootic articular surface; pro mp, medial prootic process; socc as, supraoccipital articular surface; socc-pro n, supraoccipitalprootic notch; stf, sella turcica fossa.

The parasphenoid-prootic suture runs over the anterior prootic buttress and posterior parasphenoid buttress. The suture with the parasphenoid is complex (Fig. 5), the tubera flush with the lateral wall of the prootic and laterally, the two bones contact on an oblique suture superficially. However, the prootic sockets into the parasphenoid more deeply in a “stepped tongue in groove joint” (Jones et al., 2011).

There is a clear separation between the basipostsphenoid and prootic of about 260 µm, contra Laurin (1998, p. 769). The two bones only contact in a few points anteriorly, but there is a clear sutural mark on the basipostsphenoid leaving a sub-rhomboid impression on the basipostsphenoid.

The prootic abuts the supraoccipital dorsally, becoming a broader contact ventrally where both bones are excavated to house the floccular fossa (sensu Sigogneau-Russell, 1989, Fig. 71), but named subarcuate fossa according to Olson (1938).

The prootic contacts via an interdigitating suture on the medial extension on the dorsal surface of the opisthotic (Fig. 4A), becoming looser posteriorly. A fused opisthotic and prootic has been described for *Arctognathus* (Kammerer, 2015, p. 48) and may be an ontogenetic feature.

The prootic has a dorsal supraoccipital-prootic notch, sloping to the anterodorsal process of the prootic. Between the anterodorsal process and the pila antotica there is an irregularly shaped notch, the prootic fenestra of Kemp (1969). A shallow depression covers most of the prootic lateral surface, extending to the basal region. The anterior prootic notch is a deep excavation located on the anterior surface of the prootic, medial to the contact surface of the parasphenoid-basipresphenoid and ventral to the pila antotica. The two prootics contact each other medially, within the medullary cavity, and the contact is subtriangular in sagittal cross-section. The dorsal surface of the medial prootic process forms the dorsum sella of Kemp (1969; see also Sigogneau-Russell, 1989). This is probably the same as the so-called basicranial process of the periotic of Olson (1944).

In posterior view, on the basal region of the prootic, there is a subtriangular fossa formed by the posterior crest of the medial prootic process, by the medial wall of the prootic and bordered ventrally by the dorsal surface of the basipostsphenoid (this study). A mediolaterally-oriented foramen, the facial foramen, pierces the lateral wall near the basal region of the prootic and exits at the base of the medial prootic process. This foramen has ∼600 µm diameter laterally and ∼400 µm at its mid-section.

There is no shallow depression posteroventral to the base of the anterodorsal process on the right prootic (contra Laurin, 1998); however, on the left prootic there is indeed a shallow depression. Due to the asymmetry of this feature, differences may be the result of taphonomic distortion.

### Basioccipital

**Figure 6:**
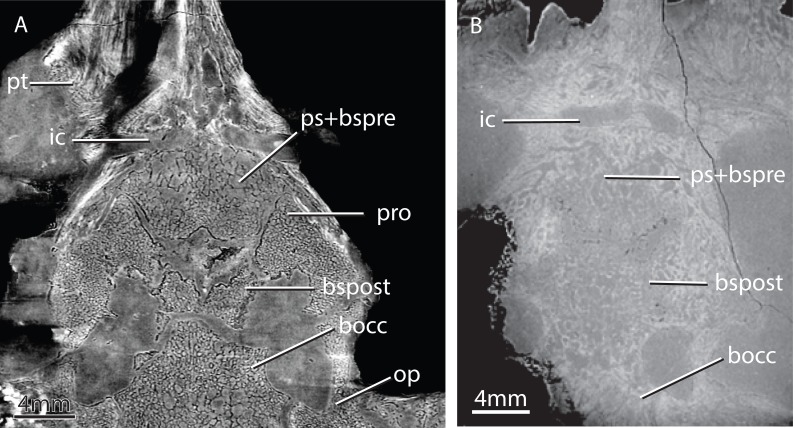
Horizontal virtual sections of the skull of various gorgonopsian basicrania at different ontogenetic stages. Notice, for example, the wide separation between the basipostsphenoid and the parasphenoid-basipresphenoid complex in GPIT/RE/7124 (A) versus the condition in GPIT/RE/7119 (B). Abbreviations: bocc, basioccipital; bspost, basipostsphenoid; ic, internal carotids; op, opisthotic; ps+bspre, parasphenoid-basipresphenoid; pro, prootic; pt, pterygoid.

The basioccipital forms the ventral border of the foramen magnum. The basioccipital contacts the exoccipital dorsolaterally, the opisthotic laterally and the basipostsphenoid anteriorly (Figs. 2 and 3). The parasphenoid-basipresphenoid does not contact the basioccipital (contra Laurin, 1998, Fig. 5; Parrington, 1955, Figs. 6 and 10). The articulation facet with the exoccipital is ellipsoidal in shape and dipping posteriorly. A clear separation between basioccipital and basipostsphenoid is discernible dorsally, but the two bones become co-ossified ventrally (Fig. 6). The separation between the opisthotic is conspicuous (distance between the bones is 200–300 µm), forming a ball-and-socket joint (Figs. 4 and 6). The articulation facet with the opisthotic is ellipsoidal, with the major axis dipping anteriorly.

**Figure 7:**
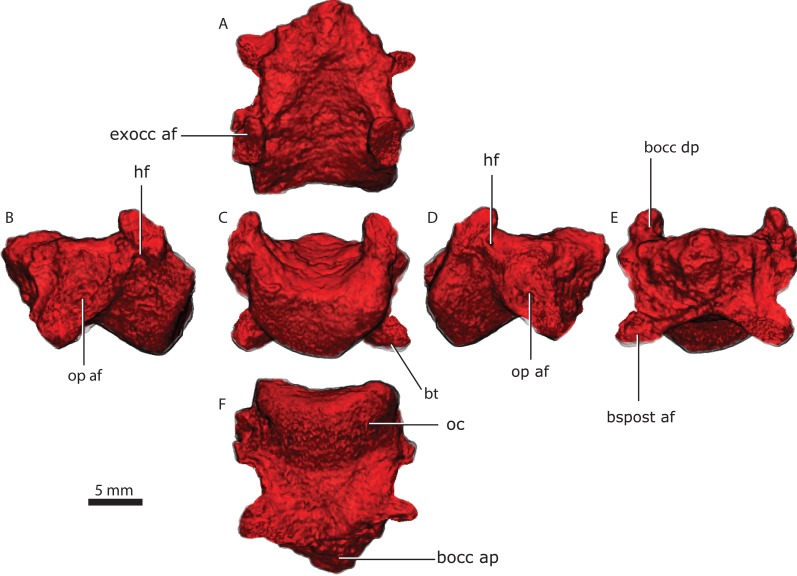
Basioccipital in dorsal (A), lateral left (B), posterior (C) lateral right (D), anterior (E), and ventral (F) views. Abbreviations: bocc ap, anterior process of the basioccipital; bocc dp, dorsal process of the basioccipital; bspost af, basipostsphenoid articular facet; bt, basal tubera; exocc af, exoccipital articular facet; hf, hypoglossal foramen; oc, occipital condyle; op af, opisthotic articular facet.

The occipital condyle is reniform in shape, possessing a shallow median depression on its dorsal surface (Fig. 7C). In ventral view, the occipital condyle is somewhat V-shaped (Fig. 7A). The articulation with the exoccipital is formed by a short dorsal process, which has an oblique orientation relative to this bone (Figs. 7B and 7F). The dorsal process slopes anteriorly into a shallow crest that meet its counterpart on the anterior tip of the basioccipital. The anterior part of the basioccipital forms a short, triangular-pyramidal process (Figs. 7A, 7E, 7F). The dorsal process of the basioccipital is pierced by the hypoglossal foramen (Figs. 7B and 7D), which served for the passage of the hypoglossal nerve (cn XII). The hypoglossal foramen is a horizontally-oriented canal with 600–800 µm in diameter. The basioccipital forms the posteriormost part of the basal tubera, which continues onto the basipostsphenoid and parasphenoid. An ellipsoidal foramen perforates the ventral surface of the contact between the basioccipital and basipostsphenoid.

### Exoccipital

**Figure 8:**
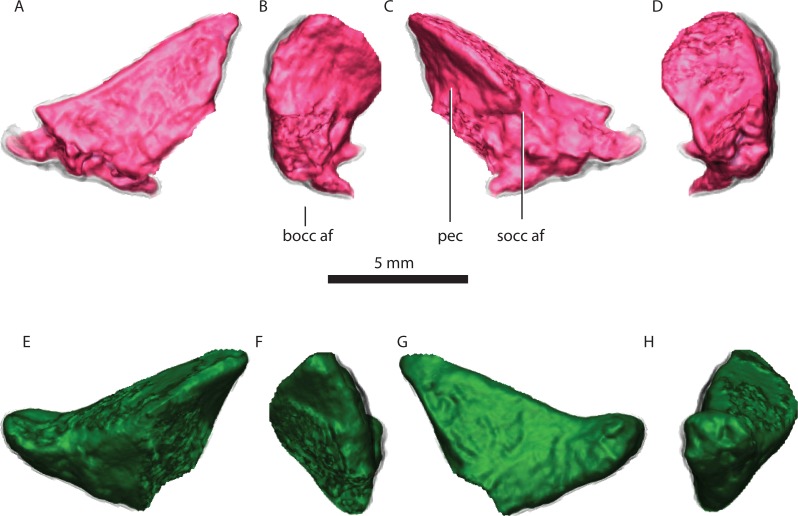
Right exoccipital in posterior (A), medial (B), anterior (C), lateral (D) views. Left exoccipital in anterior (E), medial (F), posterior (G), lateral (H) views. Abbreviations: bocc af, basioccipital articular facet; pec, pyramidal exoccipital crest; socc af, supraoccipital articular facet.

The exoccipital has the typical subtriangular shape in posterior view described by Kemp (1969). It forms part of the lateral wall of the foramen magnum and contacts the basioccipital ventrally along its medial edge (Fig. 2A). The basioccipital is partially co-ossified to the exoccipital ventrally, but there is a clear separation dorsally between the two bones on the tomographs. The exoccipital does not contact the opisthotic. The exoccipital contacts the supraoccipital along its dorsal edge. The dorsal edge is sinusoidal on the right exoccipital but almost straight on the left. The posterior surface and the ventral margin of the exoccipital together with the ventromedial corner of the supraoccipital constitute the dorsal border of the jugular foramen (Fig. 2A). The exoccipital does not form part of the occipital condyle (contra Kemp, 1969, p. 18). Kemp (1969, p. 19) describes a “small pyramidal exoccipital process”. This process is probably best described as the pyramidal exoccipital crest that results from the ventral facet with the basioccipital and dorsal facet with the supraoccipital (Fig. 8).

The exoccipital forms part of the anterior and dorsal wall for the passage of the glossopharyngeal and the vagoaccessory nerves (cn IX, X, XI), see also Colbert (1948).

The exoccipital prevents the supraoccipital from contacting the basioccipital, although that element extends far ventrally.

### Opisthotic

**Figure 9:**
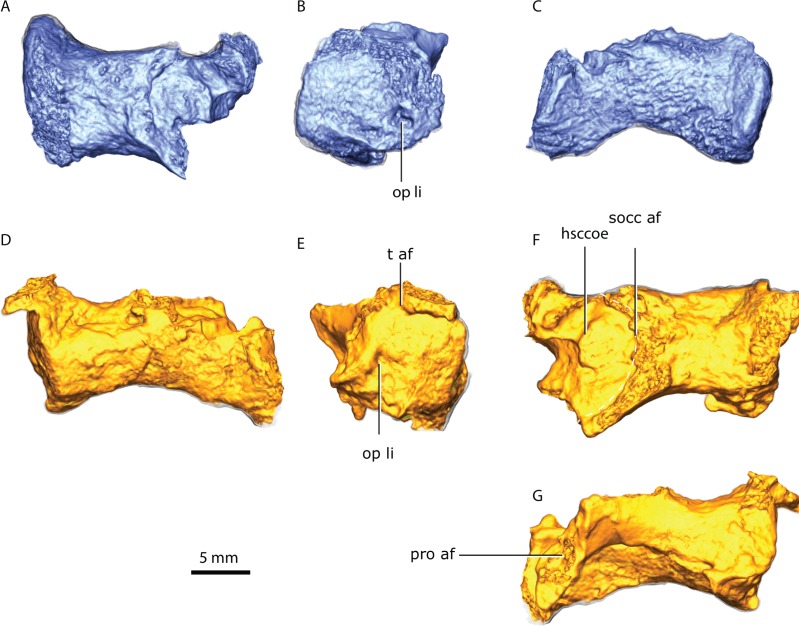
Right opisthotic in dorsal (A), lateral (B) and posterior (C) views. Left opisthotic in posterior (D), lateral (E), dorsal (F) and anterior (G) views. Abbreviations: hsccoe, osseous enclosure of the horizontal semicircular canal; op li, lateral incisure of the opisthotic; pro af, prootic articular facet; socc af, supraoccipital articular facet; t af, tabular articular facet.

The opisthotic is a rod-like bone that contacts the supraoccipital dorsally, the basioccipital medially, the tabular on its posterolateral extremity, the squamosal on its anterolateral extremity and the prootic anteriorly (Figs. 2 and 9). The ventral margin of the opisthotic is strongly concave, compared with its gently embayed dorsal margin. The opisthotic forms the ventral margin of the posttemporal fenestra and it contributes to the ventral margin of the jugular foramen on its anterodorsal extremity (Fig. 2). The opisthotic and supraoccipital are firmly co-ossified, leaving no sutural marks (Fig. 4E).

The anterior surface of the opisthotic is dominated by an anteriorly-directed process that progressively thickens medially, serving as the posterior and lateral support of the prootic (Fig. 9).

The lateral surface of the opisthotic is subcircular, and its gently convex lateral margin is carved by a lateral incisure (Figs. 9B and 9E). In cross-section, the opisthotic is subovoid at its median section, and subrectangular medially.

### Supraoccipital

**Figure 10:**
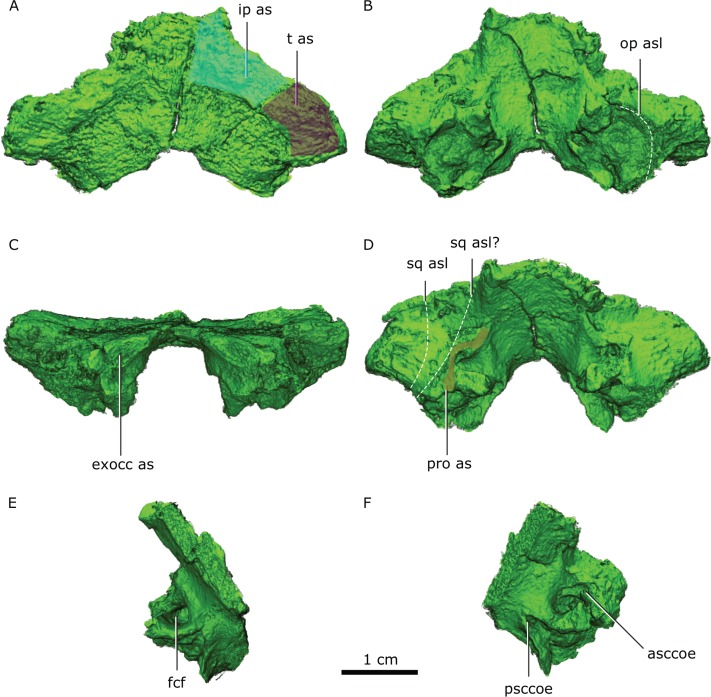
Supraoccipital in dorsal (A), ventral (B) posterior (C) and anterodorsal (D) views. Right half of the supraoccipital in medial (E) view. Left half of the supraoccipital in anteroventral (F) view. Abbreviations: asccoe, anterior semicircular canal osseous enclosure; exocc as, exoccipital articular surface; fcf, flocular complex fossa; ip as, interparietal articular surface; op asl, opisthotic articular surface limit; pro as, prootic articular surface; psccoe, posterior semicircular canal osseous enclosure; sq asl, squamosal articular surface limit as with the preserved portion of the squamosal and possible articular limit if the squamosal was entirely preserved; t as, tabular articular surface.

The supraoccipital is a rather complex element, however only its posterior occipital exposure is typically described (Fig. 10). The supraoccipital is a single median element (Fig. 4B). The subrectangular posterior exposure of the supraoccipital is only a small portion of the bone which extends significantly further dorsally but is covered by the tabular and interparietal in posterior view (Fig. 2).

The supraoccipital is comprised of an alar region as well as the supraoccipital body (Fig. 10). The supraoccipital body is a complex stout ventral structure that sutures with the prootics anteriorly, with the opisthotics ventrolaterally, and the exoccipitals posteriorly at its most ventromedial part (Fig. 2). The alar region is wedged along its dorsal extension between the tabulars anteriorly and the squamosals posteriorly (Fig. 10).

The supraoccipital body is a subrectangular buttress extending mediolaterally that encompasses: the anterior process (Kemp, 1969, Fig. 22B), the floccular fossa, the foramen for the posterior semicircular canal and the horizontal semicircular canal, and constitutes the articular facet for the prootic, opisthotic and exoccipital (Fig. 10).

The anterior process projects dorsally from the medial surface of the supraoccipital body, forming the ventral margin of the floccular fossa and the base for the prootic suture (Figs. 10C, 10D). The prootic sutural facet is T-shaped rotated medially, with the base of the “T” being the anterior process. The suture with the opisthotic is a laterally-rotated U-shape, forming a deep fossa between the posterior surface of the opisthotic and the anterior surface of the supraoccipital (Figs. 10E, 10J).

The supraoccipital forms the dorsal border of the foramen magnum, forming the ventral supraoccipital embayment (Fig. 2). An emargination on the ventrolateral edge of the supraoccipital alar region forms the dorsal margin of the posttemporal fenestra (Fig. 2).

### Basipostsphenoid

**Figure 11:**
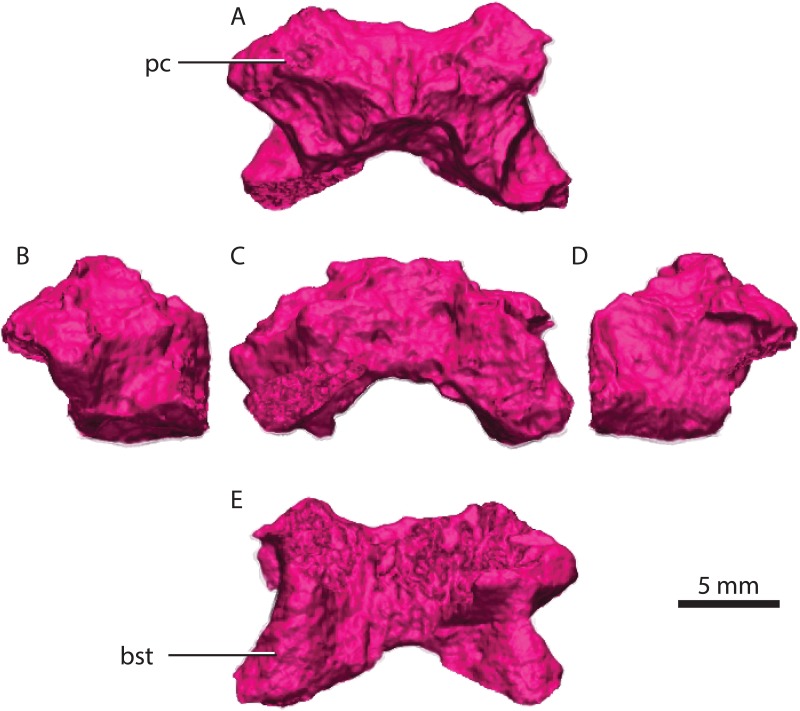
Basipostsphenoid in dorsal (A), lateral left (B), posterior (C), lateral right (D), ventral (E) views. Abbreviations: bst, basisphenoidal tubera; pc, parabolic crest.

The basipostsphenoid is undeformed and completely preserved. It is composed of the basisphenoidal tubera on the ventral side and the subhexagonal main body (Fig. 11). The anterior and posterior margins of the basipostsphenoid main body are concave.

A sheath of bone projecting posteriorly from the parasphenoid covers nearly half of the ventral surface of the basipostsphenoid (Fig. 11). On each side of the dorsal surface of the basipostsphenoid there is a parabolic shaped crest that develops from the lateral corner towards its median section and inflects posteriorly towards the posterior corner of the basipostsphenoid (Fig. 11A).

### Parasphenoid-basipresphenoid

The parasphenoid-basipresphenoid is pristinely preserved and it is only slightly plastically distorted as a result of mediolateral shear. The parasphenoid-basipresphenoid contacts the pterygoid along the parasphenoid rostrum anteriorly. Along its posterior border, the parasphenoid-basipresphenoid contacts the basipostsphenoid on the ventral half, and the prootic on the dorsal half. The interdigitating suture between the parasphenoid-basipresphenoid and basipostsphenoid is difficult to extricate, however, the separation between the prootic and parasphenoid-basipresphenoid is evident in the tomographs (Fig. 6).

**Figure 12:**
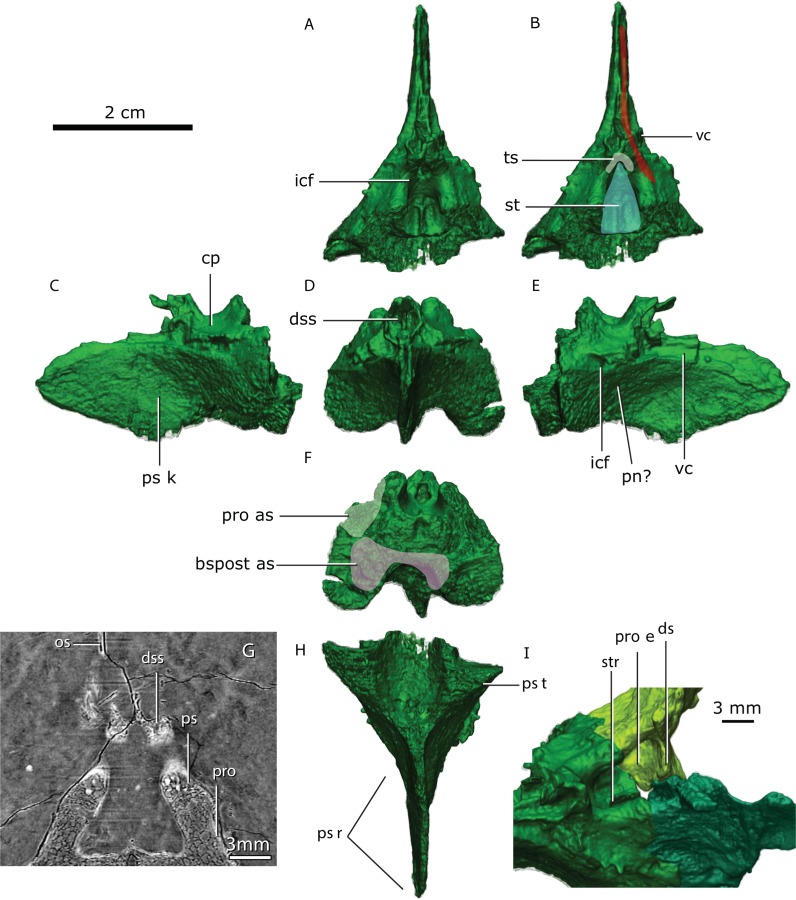
Co-ossified parasphenoid and basipresphenoid in dorsal (A, B), lateral left (C), anterior (D), lateral right (E), posterior (F), ventral (H) views and in articulation with the prootic in anterodorsal view (I), tomographic horizontal slice showing the dome-shape structure and the suture between the parasphenoid + basipresphenoid and prootic (G). Abbreviations: bspost as, articular surface for the basipostsphenoid; cp, clinoid process; ds, structural dorsum sella; dss, dome-shaped structure; icf, internal carotid foramina; os, orbitosphenoid; pn, palatine nerve; pro, prootic; pro as, articular surface for the prootic; pro e, prootic embayment; ps, parasphenoid; ps k, parasphenoid keel; ps r, parasphenoidal rostrum; ps t, parasphenoidal tubera; st, sella turcica; str, sella turcica ridge; ts, tuberculum sella; vc, vidian canal.

The parasphenoidal tubera are the most prominent features in the ventral view of the parasphenoid (Figs. 3, 12C, 12G). The parasphenoidal tubera connect to the parasphenoid rostrum anteriorly via the anterior parasphenoidal lamina (Fig. 12G), and to the basisphenoidal tubera via the posterior parasphenoidal lamina (Figs. 3, 12G). The parasphenoidal tubera are somewhat triangular in shape and are significantly larger than the basisphenoidal tubera (Figs. 11 and 12). The posterior parasphenoid fossa (Fig. 12) is a deep excavation delimited medially by the prootic and basipostsphenoid suture and by a crest that converges to the parasphenoidal tubera laterally. There is no basipterygoid process (contra Olson, 1944, Fig. 20).

The parasphenoid rostrum is deepest at the intersection of the right and left anterior parasphenoidal lamina (Fig. 12C). In lateral aspect, the ventral edge is slightly concave whereas the dorsal edge is convex, giving the rostrum a subtriangular shape. Two thin crests on the dorsal edge of the parasphenoid rostrum form a trough (the vidian canal) on its posterior portion that meet at midlength (Figs. 12A, 12C).

On its dorsal side, the sella turcica is delimited laterally by the two saddle-shaped dorsal buttresses of the parasphenoid-basipresphenoid (the processus clinoideus?), and by the anterior prootic buttress as well as the medial prootic process posteriorly (contra Sigogneau-Russell, 1989, who described the sella turcica as part of the “basisphenoid”). The sella turcica is divided in two ellipsoidal depressions separated by a short ridge (as in Olson, 1944; Sigogneau-Russell, 1989). The sella turcica is deeper posteriorly and becomes shallower anteriorly as the parasphenoid dorsal buttress slopes ventrally (Figs. 12A, 12H).

A horizontal trough, the vidian canal, separates the posterior parasphenoid buttress from the parasphenoid keel (Fig. 12E). On the posterior portion of the parasphenoid-basipresphenoid a medioanteriorly-directed foramen (∼600 µm) pierces the lateral surface of the parasphenoid-basipresphenoid at the level of the horizontal trough, the cerebral branch of the internal carotid (Fig. 12E). This foramen is L-shaped and exits the dorsal surface of the parasphenoid-basipresphenoid on a deep fossa anterior to the sella turcica, the hypophyseal fossa plus the exit for the cerebral branch of the internal carotids (Fig. 12A). Located anterior to the hypophyseal fossa, there are posteriorly-convex dome-shaped structures, separated by a median anteriorly-directed process that has been undescribed before, possibly a remnant of the orbitosphenoid (Fig. 12D).

### Orbitosphenoid

**Figure 13:**
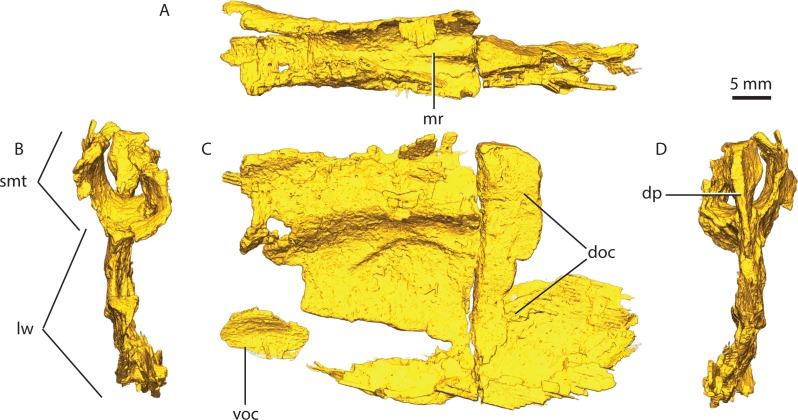
Orbitosphenoid in dorsal (A), posterior (B), lateral (C) and anterior (D) views. Abbreviations: doc, dorsal ossification center; dp, dorsal process; lw, lateral wall; mr, median ridge; smt, semi-tubular region of the orbitosphenoid; voc, ventral ossification center.

Although the orbitosphenoid is nearly complete, it is lacking part of the lateral wall posteriorly and there is a dorsoventral crack traversing its anterior portion (Fig. 13). Olson (1944, p.76) described the orbitosphenoid as laying in the dorsal groove of the parasphenoid, but although it is not as well preserved in this region it does not reach the parasphenoid.

The orbitosphenoid is a semi-cylindrical bone articulating with the frontal and parietal dorsally and continues as a lateral wall ventrally from the sagittal axis of the skull (Fig. 13B). At the intersection between the semi-cylindrical and lateral wall regions of the orbitosphenoid, two parallel internal cavities extend along the posterior section of the bone (Fig. 13A).

The ventral region of the tubular region is smooth posteriorly, however, a median ridge raises at about midlength of the frontal (Fig. 13A). The median ridge becomes progressively taller and more acute, eventually forming a distinct separation between the two lobes of the olfactory bulb (Fig. 13D). On its anteriormost zone, the median ridge forms a distinct dorsally-inflated process that articulates with the sagittal suture of the frontals.

### Tabular

**Figure 14:**
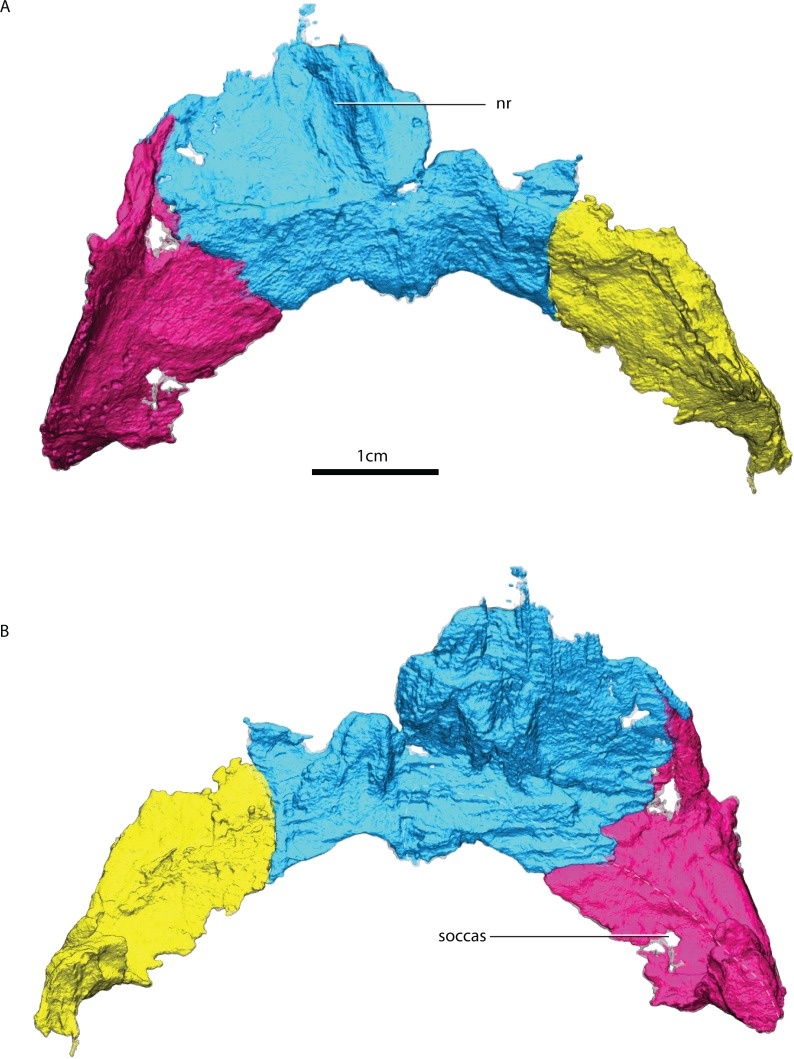
Renderings of the tabulars and interparietal. Left and right tabulars and the interparietal in posterior (A) and anterior (B) views. Abbreviations: nr, nuchal ridge; socc as, supraoccipital articular surface.

The tabular is subtriangular in shape with a raised lateral edge for articulation with the squamosal (Figs. 2 and 14). The left tabular is well preserved, but the dorsal section of the right tabular is missing (Fig. 14). The tabular contacts the interparietal along its dorsal surface (Fig. 14). The left tabular is firmly sutured to the wing of the interparietal and it is very difficult to separate them on the basis of the tomographs. In this case the external surface allows a better interpretation of the sutures. Most of the anterior surface of the tabular makes the articular facet for the supraoccipital (Fig. 14B). The two tabulars are separated in the sagittal plane of the skull by the interparietal (Fig. 14).

### Interparietal

The interparietal is incompletely preserved with the right wing being significantly incomplete. There is a break between the more robust median section of the interparietal, comprising the nuchal crest, and the left wing (Fig. 14). The nuchal crest bulges slightly more dorsal than the ventral border of the interparietal and tapers dorsally. There is a small foramen that traverses mediolaterally the interparietal wing away from the nuchal crest (Fig. 4I). The suture between the interparietal and the tabular is hard to discern using the tomographs.

### Squamosal

**Figure 15:**
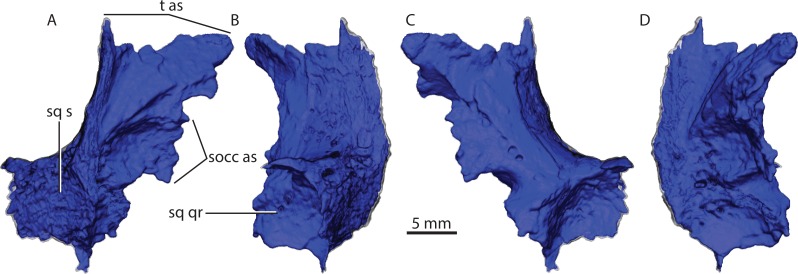
Left squamosal in posterior (A), lateral (B), anterior (C) and medial (D) views. Abbreviations: socc as, supraoccipital articular surface; sq qr, quadrate recess of the squamosal; sq s, squamosal sulcus; t as, tabular articularsurface.

Only the dorsal ramus of the squamosal is preserved in both sides, thus missing the zygomatic portion (Figs. 2, 15A). The preserved squamosal contacts the tabular posteriorly and the supraoccipital medially. The articular surface for the tabular extends dorsoventrally along a vertical crest delimiting its lateral border and flares anteriorly into an elongated subtriangular process (Fig. 15). Part of the squamosal sulcus is preserved in posterior view forming a flat area posteriorly (Fig. 4I). The supraoccipital articular facet forms an embayment on the surface of the squamosal delimited by a parabolic crest (Figs. 15A and 15D). The quadrate recess of the squamosal is a deeply excavated depression delimited posteriorly and dorsally by a subcircular crest (Fig. 15B).

### Brain endocast

**Figure 16:**
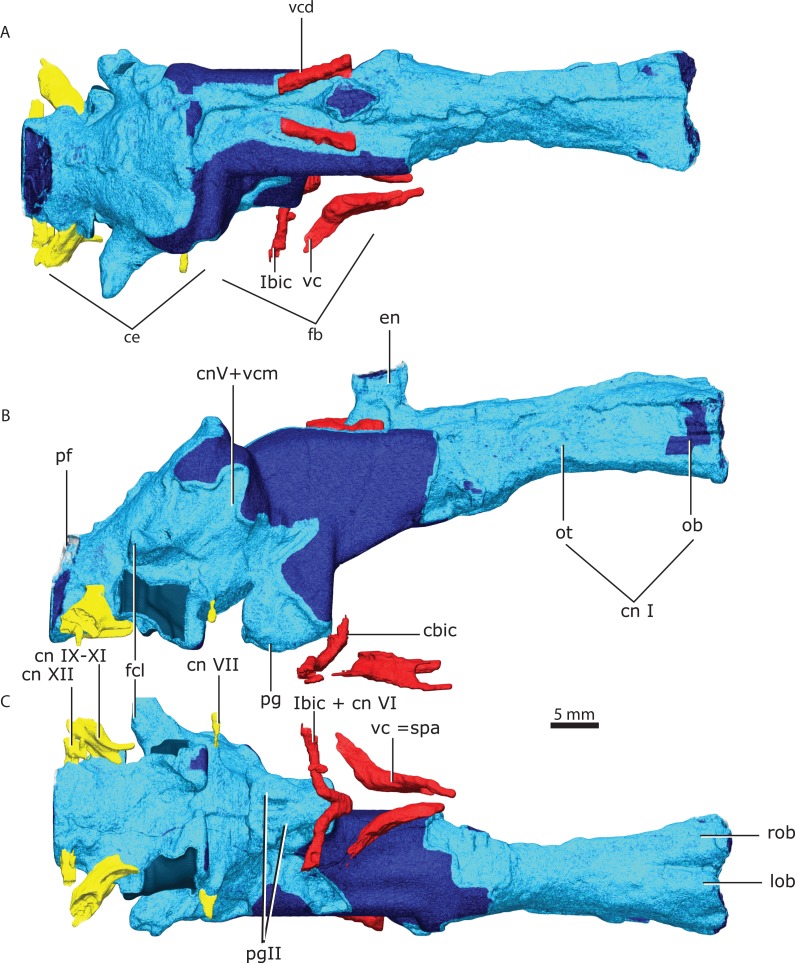
Brain endocast in dorsal (A), lateral (B), ventral (C) views. Parts in light blue indicate direct contact with surrounding bones, unlike parts in darker blue. Abbreviations: ce, cerebellum; cnI, olfactory nerve; cnV +vcm—trigeminal nerve and vena capitis medialis; cnVI, abducens nerve; cnVII, facial nerve; cnIX-XI, glossopharyngeal and vagoaccessory nerves; cnXII, hypoglossal nerve; en, epiphyseal nerve; fb, forebrain; fcl, floccular complex lobes; ibic, internal branch of the internal carotid; lob, left olfactory bulb; ob, olfactory bulb; ot, olfactory tract; pg, pituitary gland; pgll, pituitary gland lateral lobes; pf, pontine flexure; rob, right olfactory bulb; vc, vidian canal; vc=spa, vidian canal where the sphenopalatine artery passes; vcd, vena capitis dorsalis.

Three sections of the skull offer reliable proxies of the brain endocast anatomy. However the occipital region is slightly laterally displaced relative to the skull roof and orbitosphenoid, hampering a good fit between the olfactory tract region and the hindbrain (Figs. 1 and 16). The region encased by the ventral surface of the semi-cylindrical region of the orbitosphenoid bounds the cast of the olfactory bulbs and tract as well as part of the forebrain. The region enclosed by the median contact of the two parietals (pineal foramen), embrace the cast of the epiphyseal nerve. The posterior section of the skull delimited by the parasphenoid-basipresphenoid, prootics, supraoccipitals, exoccipitals and opisthotics bounds the cast of the mid- and hindbrain (Fig. 1).

Although the olfactory bulbs are large, the cerebellum is still more expanded than the cerebrum (Fig. 16). The olfactory bulbs are connected to the forebrain by the olfactory tract. The olfactory bulbs are divided anteriorly by the median ridge. The orbits are located at the level of the olfactory bulbs. The connection between the cerebrum and the epiphyseal nerve is not clear because the orbitosphenoid shifted laterally relative to the parietals. However, the anterior portion of the cerebrum has an oblate ellipsoidal volume that is truncated anteriorly by the olfactory tracts.

The endocast bounded by the ventral surface of the supraoccipitals enclose symmetrical domes on the brain that may be divided by an interhemispheral fissure at the level of the sagittal supraoccipital suture (Fig. 16A).

The floccular complex lobes project posterolaterally from the cerebellum and arch dorsally (Fig. 16A). The floccular complex lobes are solely delimited by the supraoccipital, however there is an embayment on the dorsal portion of the prootics that forms a lateral inflation of the cerebellum that connects posteriorly with the floccular complex lobes (Figs. 16A, 16B). The total volume of the brain is ∼6,767 mm^3^.

A clear division between the forebrain and the midbrain is marked by an isthmus (Fig. 16A). The only distinguishable structure of the ventral midbrain is the hypophysis (or pituitary gland) as the optic lobes are not distinct from the hindbrain (Fig. 16B). The hypophysis is delimited by the medial process of the prootics posteriorly, and anterolaterally by the dorsum sella, forming a broad subcylindrical structure (Fig. 16B). The hypophysis is divided ventrally into two laterally-positioned pituitary lobes (Fig. 16C).

The pontine flexure marks the separation between the hindbrain and the medulla oblongata and is located posteriorly to the floccular complex lobes (Fig. 16B), contrary to the condition in dicynodonts (Castanhinha et al., 2013).

### Cranial nerves and vasculature

The epiphyseal nerve (diameter between ∼2,000 and 2,300 µm) exits dorsally through the pineal foramen, embraced by the parietals (Fig. 16A) and a small portion of the inferred path vena capitis dorsalis is preserved in the ventral surface of the parietal and borders the posterior half of the epiphyseal nerve (Fig. 16A). All other preserved cranial nerves exit from the ventral side of the brain (endocast) except the trigeminal nerve that exits at about mid-height of the brain (Fig. 16B) along with the vena capitis medialis between the pila antotica and the anterodorsal process of the prootic. The abducens nerve (cn VI) probably had the same path as the cerebral branch of the carotid artery (Fig. 16C). The internal carotids pierce the parasphenoid-basipresphenoid laterally and join in the median part of the skull and exits anterior to the sella turcica (Fig. 16B). This path joins with the vidian canal that runs along the laterodorsal side of the parasphenoid-basipresphenoid complex (Figs. 16B, 16C). A small caliber canal (diameter: ∼385 µm) pierces the lateral wall of the parasphenoid- basipresphenoid complex ventral to the internal carotid foramen (Fig. 16B). This canal continues horizontally and bends dorsally towards the internal carotid artery. Given the conservative pattern of the hindbrain vasculature in tetrapods this canal may be the orbital artery (Rahmat & Gilland, 2014). The facial nerve (cn VII) pierces the prootic ventrolaterally oriented, and has a diameter of ∼480 µm (Fig. 16C). There is no osseous enclosure for the vestibulocochlear nerve (cn VIII) as the brain endocast contacts directly the medial wall of the osseous labyrinth (cf. Sigogneau, 1974). The vagoaccessory and glossopharyngeal nerve (cn IX–XI) is bounded by the supraoccipital dorsally, and the opisthotic and basioccipital ventrally (Fig. 16C). The vagoaccessory and glossopharyngeal nerve exit the brain laterally right anterior to the pontine flexure, and have a diameter of ∼1,400 µm. The osseous enclosure for the hypoglossal nerve (cn XII, Fig. 16C) exits the brain ventrolaterally and pierces the dorsal process of the basioccipital (diameter ∼590 µm).

## Discussion

**Figure 17:**
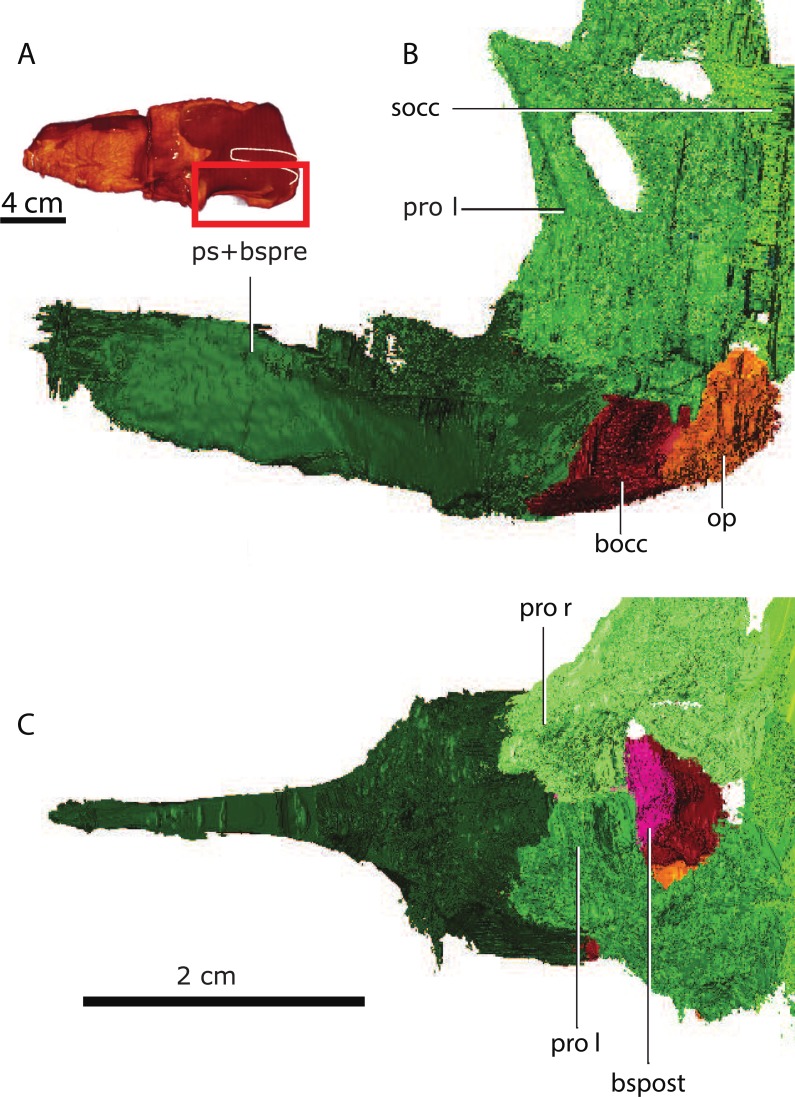
GPIT/RE/7119 cranium in lateral view (A). Segmented portions of the basicranium in (B) lateral and (C) dorsal views. Due to extensive co-ossification the more dorsal portions of the occiput the bones are impossible to extricate from each other in the tomograms. Abbreviations: bocc, basioccipital; bspost, basipostsphenoid; op, opisthotic; pro l, left prootic; pro r, right prootic; ps+bspre, parasphenoid and basipresphenoid; socc supraoccipital.

We analyzed two gorgonopsian specimens, GPIT/RE/7124 and GPIT/RE/7119, using propagation phase contrast synchrotron micro-computed tomography. Our results uncovered previously unknown anatomical features of the gorgonopsian braincase that in some aspects differ from previous descriptions. We discuss below the enigmatic posterior ossification of the basisphenoid and its possible role on developmental processes and ontogeny among synapsids. In addition, we make extensive comparisons of the basicranium and occiput of GPIT/RE/7124, GPIT/RE/7119 and other published gorgonopsian specimens (Fig. 17). Finally, we discuss implications of our endocranial reconstructions for sensory suite and head posture in gorgonopsians.

### Ontogenetic stage of GPIT/RE/7124

Although dubious for some reptiles (Bailleul et al., 2016), among synapsids skull sutural closure is a reliable indicator of ontogenetic maturity (Dwight, 1890; Todd & Lyon, 1925; Krogman, 1930; Schweikher, 1930; Chopra, 1957). Despite recent efforts to extricate ontogenetic and phylogenetic characters among gorgonopsians (Kammerer, 2016), ontogenetic patterns of character change and gorgonopsian systematics remain insufficiently understood, particularly within the basicranium. Thus, it is necessary to use alternative lines of evidence such as sutural closure or bone histology to assess a relative degree of maturity among gorgonopsians. Figure 6 shows horizontal sections of the basicranial region of two ontogenetic stages in two different gorgonopsians: GPIT/RE/7124 and GPIT/RE/7119. The sutures remain visible (e.g., basipresphenoid-basipostsphenoid, prootic-basipostsphenoid, opisthotic-basioccipital, basioccipital-basipostsphenoid) and separated in GPIT/RE/7124 (Figs. 6A and 6B), but they are co-ossified in GPIT/RE/7119. In GPIT/RE/7119, only the opisthotic-basioccipital suture is clearly visible (Fig. 17), while the rest of the sutures, although visible, are hardly distinguishable from the trabeculae mesh.

In GPIT/RE/7119, bone trabeculae are larger than in GPIT/RE/7124, indicating significant bone remodeling and resorption. It is known that the trabecular length scales with body mass (Swarts, Parker & Huo, 1998), and the larger specimen GPIT/RE/7119 (∼20 cm estimated skull length) has indeed larger trabeculae when compared to GPIT/RE/7124 (14–15 cm and skull length). The incipient sutural closure and the small trabeculae of GPIT/RE/7124 are indicative of the physical immaturity (as conceptualized by Araújo et al., 2015) of this gorgonopsian specimen.

### Comparative anatomy of the occiput

**Figure 18:**
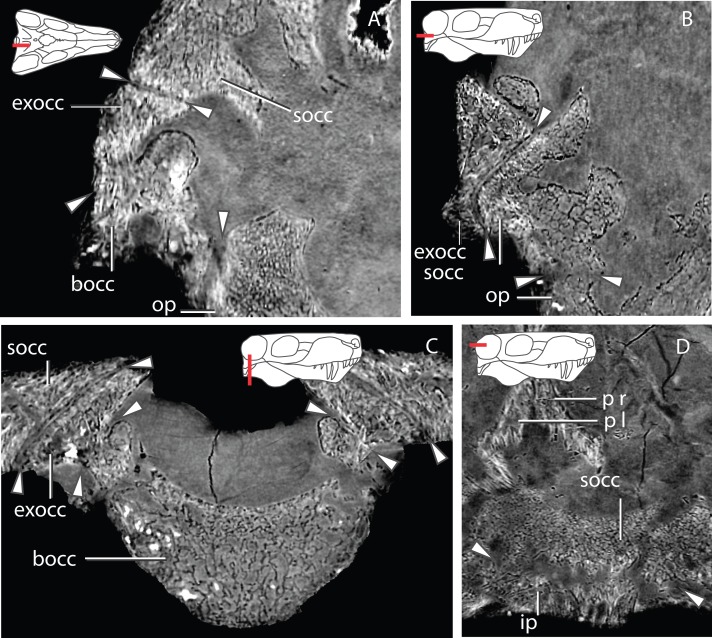
Contentious issues concerning the gorgonopsian occiput clarified by GPIT/RE7124. (A–C) show that the exoccipital does not contact the opisthotic. (A), sagittal section through the right exoccipital; (B) horizontal section through the right exoccipital, supraoccipital and opisthotic; coronal section through the basioccipital and the two exoccipitals. (D), shows the suture and overlap between the supraoccipital and interparietal. Abbreviations: bocc, basioccipital; exocc, exoccipital; ip, interparietal; op, opisthotic; p l, left parietal; p r, right parietal; socc, supraoccipital. Arrows show the locations of the sutures between bones.

The occiput is a relatively conserved region in gorgonopsians, but accurate information is often inaccessible due to co-ossification of the bone elements and preservational damage. Most specimens have a concealed suture between the exoccipital and basioccipital, but also between the opisthotic and tabular, and the supraoccipital and exoccipital (Sigogneau, 1970; Sigogneau-Russell, 1989). As a result, there is contradictory information in previous publications concerning the formation of the occipital condyle. Olson (1938) clearly states that the occipital condyle is solely formed by the basioccipital. Conversely, Kammerer (2016) posits that the lateral portions of the occipital condyle are formed by exoccipitals. Kemp (1969) describes the exoccipital as forming the ventromedial corner of the occipital condyle. In specimens where the limits are discernible, Sigogneau (1970) and Pravoslavlev (1927) depict the exoccipitals as being excluded from the occipital condyle. Sigogneau (1970) depicts this condition in four specimens (i.e., AMNH 5515, BPI 259, IGP U 28, RC 2), but does not describe the condition specifically. Unfortunately, the occipital condyle is not preserved in GPIT/RE/7119. However, in GPIT/RE/7124 there is a clear suture visible in the tomographs separating the exoccipital from the supraoccipital and basioccipital (Fig. 18). The exoccipital is completely excluded from the occipital condyle and only the ventromedial corner of the exoccipital contacts the basioccipital, somewhat resembling the condition described by Kemp (1969), except that the basioccipital has a dorsal process that prevents the exoccipital from contacting the occipital condyle. However, we do not rule out that some specimens might have some contribution of the exoccipital to the occipital condyle, particularly on its dorsal component (Christian Kammerer, pers. comm., 2016). Moreover, within the basioccipital, there is no evidence of sutures, trabeculae size variation or different types of bone (cortical versus trabecular). Importantly, there is no opisthotic-exoccipital suture, although the two bones nearly contact. These two bones are separated by the supraoccipital, despite the extension of the exoccipital lateral corner (Fig. 18). Nevertheless, this feature might be different in other specimens (Christian Kammerer, pers. comm., 2016).

GPIT/RE/7124 can clarify the supraoccipital-interparietal relationship (Fig. 18). In all serial-sectioned specimens (Olson, 1938; Kemp, 1969; Sigogneau, 1970) the supraoccipital extends dorsally, being partially covered by the interparietal. Kemp (1969, Fig. 21) and Sigogneau (1970, Figs. 23, 51, 81 and 151) depict the interparietal reaching the endocranial cavity, but in laterally-shifted sagittal sections. However, in a median sagittal section, the interparietal is superficial in a variety of specimens (e.g., BPI 277, NHMUK R 4053 and RC 57) with no contact with the endocranial cavity.

### Comparative anatomy of the basicranium

Although conservative in various traits, there is significant variation in the gorgonopsian basicranium. Notably, the two specimens here studied highlight variation that can be attributed to ontogeny. In agreement with previous reports (Olson, 1938; Kemp, 1969; Sigogneau, 1970), our analysis of GPIT/RE/7124 and GPIT/RE/7119 showed that the prootics meet medially through a medial process that overlaps the basisphenoid and basioccipital. The medial contact of the prootics is apparent in the coronal section “F” in Olson (1938). This process may be more or less robust most likely depending on the ontogenetic stage. More ontogenetically advanced gorgonopsians, such as GPIT/RE/7119, show a robust and dorsoventrally expanded medial process of the prootic (Figs. 17, 18B, 18C), whereas GPIT/RE/7124 shows a relatively feebler medial contact and consequently a shallower depression for the hypophyseal fossa (Figs. 18A, 18B).

The prootic has significant differences in GPIT/RE/7124 (as well as BPI 3, TMP256 and NHMUK R 5743, Sigogneau, 1970) when compared to the most general pattern shown by more ontogenetically advanced specimens. GPIT/RE/7119 (Figs. 17, 18B), BPI 277 (Sigogneau, 1970), NHMUK R 4053 (Sigogneau, 1970), BPI 290 (Sigogneau, 1970), RC 60 (Sigogneau, 1970), RC 34 (Sigogneau, 1970), RC 103 (Sigogneau, 1970) and UMZC T877 (Kemp, 1969) have greatly ossified prootics. Such ontogenetic differences are expected because neurocranial elements tend to ossify later in ontogeny (Koyabu et al., 2014). In ontogenetically advanced specimens, the pila antotica is not a single rod-like structure, but instead it connects to Kemp’s anterodorsal process and forms an ellipsoidal vacuity from where the trigeminal nerve and vena capitis medialis exited. However, Sigogneau (1970) and Sigogneau-Russell (1989) mistakenly identified this vacuity to form the root for the optic and oculomotor nerves. The chondrocranium in mammals has the oculomotor and trigeminal cranial nerves exiting through the same perforation of the chondrocranium (De Beer, 1937; Novacek, 1993), whereas in the reptilian outgroup the optic and oculomotor cranial nerves exit anterior to the pila antotica (De Beer, 1937; Bellairs & Kamal, 1981; Paluh & Sheil, 2013). Thus, there is no extant phylogenetic bracket supporting Sigogneau’s (1970) and Sigogneau-Russell’s (1989) views on the configuration and identity of the cranial nerves exiting the ellipsoidal prootic vacuity. To accept Sigogneau’s (1970) configuration would imply that the anterior osseous border of the vacuity is an ossification of the planum supraseptale, and hence the orbitosphenoid. However, the anterior border of the vacuity is undoubtedly bounded by the prootic.

Probably due to poor preservation of the basicranium, the pila antotica bone identity was not clear in UCMP 42701 (Laurin, 1998). Laurin (1998) stated that the pila antotica is made from a composition of various bones without specifying which. However, it is clear from the specimens studied here that the pila antotica (or Kemp’s “antero-ventral” process) is part of the prootic (Figs. 6 and 18). Nevertheless, the ossification of the pila antotica itself is unusual, and more so as part of the prootic, as it has been consistently reported in the gorgonopsian literature (Olson, 1944; Kemp, 1969; Sigogneau, 1970; Sigogneau-Russell, 1989), as well as in various other non-gorgonopsian synapsids (e.g., Boonstra, 1968; Cluver, 1971; Fourie, 1974). However, the chondrocranial pila antotica is part of the basisphenoid in various amniotes (Paluh & Sheil, 2013), including cynodonts (Crompton & Museum, 1958; Presely & Steel, 1976). It is possible that the pila antotica may be part of the prootics, but there is a clear suture between the parasphenoid-basipresphenoid and the prootics, particularly visible in horizontal view (Fig. 6). The homology/presence of the pila antotica still requires further research through morphological and evolutionary interpretation of the braincase elements in more basal synapsids, as there is contradictory evidence on pila antotica development in non-therapsid synapsids (see Olson, 1944 versus Boonstra, 1968). Meanwhile, the nomenclature/homology used by previous workers remains undisputed.

The prootic anterodorsal process does not contact the orbitosphenoid in GPIT/RE/7124 or GPIT/RE/7119 (Fig. 17), contrary to Kemp’s (1969) interpretation for *Arctognathus*. Kemp (1969) homologized the anterodorsal process to the taenia marginalis, or the parietal plate using mammalian nomenclature. The taenia marginalis is the chondrocranial connection between the otic capsule and the planum supraseptale (Paluh & Sheil, 2013). Although the anterodorsal process is topologically located on the dorsal aspect of the prootic bone (i.e., of otic capsule origin), it does not contact any osseous expression of the chondrocranium anterior domain. Thus, the argument presented by Kemp (1969) and followed by Sigogneau-Russell (1989) to explain the homology of the anterodorsal process is questionable.

Importantly, there is a significant ontogenetic signal concerning the morphology and relative size of the basipostsphenoid. In GPIT/RE/7124, the basipostsphenoid is a relatively important component of the basicranial axis, with nearly half of the basioccipital length. However, the basipostsphenoid is a minute element completely enveloped by the medial process of the prootics dorsally and the basioccipital ventrally in GPIT/RE/7119. In addition, the high degree of fusion of the basioccipital and basipresphenoid renders the interpretation in such ontogenetically advanced specimens difficult. Nevertheless, the clearly anterior suture of the basipostsphenoid and basioccipital in GPIT/RE/7199 (Fig. 17), together with the more posterior larger trabeculae and the more spongious nature of the bone, are indications for the separation of these bones. A similar configuration to what was observed in GPIT/RE/7119 (Fig. 17) was described by Olson (1938, slice “E”).

The cerebral branch of the internal carotids has a consistent route in GPIT/RE/7124 and GPIT/RE/7119 and it seems to be invariable throughout ontogeny. The cerebral branch of the internal carotids pierces the wall of the parasphenoid from each side and converges medially, then perforates the dorsal surface of the basipresphenoid as a single canal onto the sella turcica. Olson (1938) correctly identified the internal carotids in the slice “G” and demonstrated that they pierce the lateral wall of the parasphenoid (see “F” slice) but failed to show their medial convergence. Kemp (1969, Fig. 7) is in accordance with our results.

**Figure 19:**
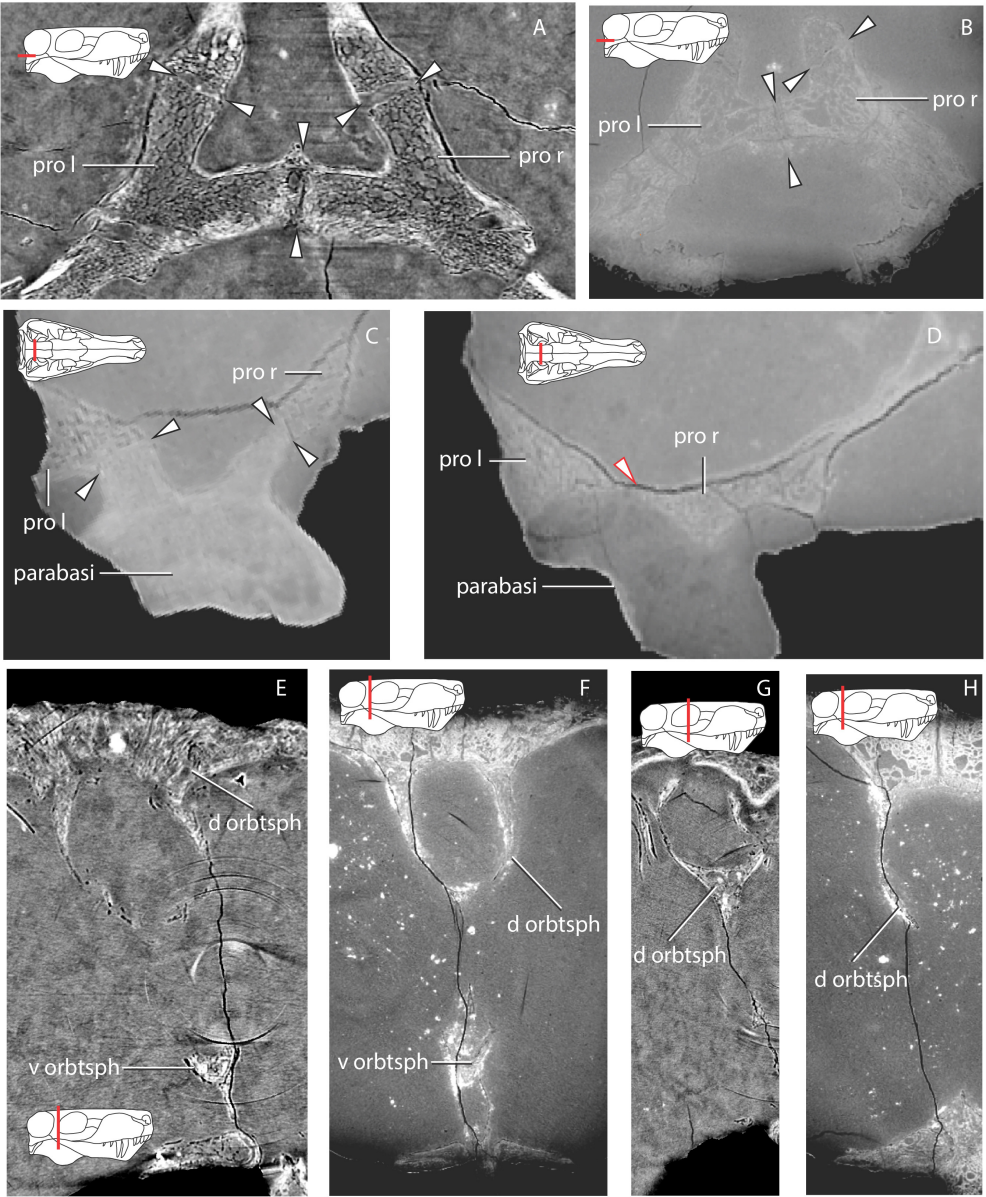
Comparisons of virtual cross sections through the basicranium. (A) GPIT/RE/7124 horizontal section through the prootics; (B) GPIT/RE/7119 horizontal section through the prootics at a similar location of (A); (C) coronal section through the prootics and parabasisphenoid of the therocephalian GPIT/RE/7139; (D) more posterior coronal section through the prootics (the parabasisphenoid is not preserved in this region, except for a small splinter of bone); the ventral ossification center of the orbitosphenoid and the dorsal ossification center in GPIT/RE/7124 and GPIT/RE/7119 in (E) and (F) respectively; and in a more anterior coronal section only the dorsal ossification center of the orbitosphenoid in GPIT/RE/7124 and GPIT/RE/7119 in G and H, respectively. Black arrows indicate the sutures between the bones and the red arrow the contact between the two prootics. Abbreviations: pro l, left prootic; pro r, right prootic; ps+bspre: parasphenoid and basipresphenoid; os doc, dorsal ossification center of the orbitosphenoid; os voc, ventral ossification center of the orbitosphenoid.

We concur with the observations of Olson (1944), Kemp (1969) and Sigogneau-Russell (1989) that the orbitosphenoid has two distinct ossified regions: a “postero-ventral ossification” laying on the parasphenoid and an “antero-dorsal”, which is continuous with the orbitosphenoid. These ossifications are indeed separate in GPIT/RE/7124. In this specimen, the “postero-ventral” ossification is a small portion of bone anterior to the internal carotid canal on the sella turcica, with a dome-shaped structure (Fig. 12). The “presphenoid” of Sigogneau (1970) should be regarded as the “postero-ventral ossification” of the orbitosphenoid. However, the more ontogenetically mature specimen GPIT/RE/7119 shows that the two ossifications are connected anteriorly but they arise from two different ossification centers (Figs. 17, 19E–19H).

### The evolution of the synapsid basicranial axis: parabasisphenoid, prootic, basioccipital

The degrees of variation and phylogenetic signal of the basicranium remain unexplored in synapsids (Rougier & Wible, 2006). We here compile and summarize the current knowledge on the evolution of the parabasisphenoid, prootic and basioccipital complex as these bones mark a key transition between the neural crest/mesoderm derived bones. It is clear, however, that further resesrch is needed, as the anatomy of the basicranium is only known from few specimens of the many synapsid groups.

In the parareptilian outgroup, the parabasisphenoid seems to be a single element contacting the basioccipital posteriorly (Spencer, 2000; Tsuji, 2013). However, within basal reptilians the prootics do not meet medially in the procolophonids *Leptopleuron* (Spencer, 2000) and *Procolophon* (Carroll & Lindsay, 1985), the captorhinid *Eocaptorhinus* (Heaton, 1979), and the basal neodiapsid *Youngina* (Gardner et al., 2010). The pareiasaur *Deltavjatia* appears to be an exception in displaying a medial contact (Tsuji, 2013), but it is possible it may result from post-mortem deformation. Extant reptilians also do not possess a medial contact between the prootics (Oelrich, 1956; Iordansky, 1973; Gaffney, 1979).

Among synapsids, the sphenacodontian *Dimetrodon* has a fused parabasisphenoid that contacts directly the basioccipital (Romer & Price, 1940; Brink & Reisz, 2012). The prootics meet medially, similarly to the condition in the gorgonopsians forming a structural dorsum sella (Romer & Price, 1940). This dorsum sella formed by the prootics is not homologous to the human dorsum sella, from which the term originally derived (Boele, 1828), but it is a structural dorsum sella in the sense that it forms the posterior wall of the hypophyseal fossa. Among therapsids, the burnetiamorph biarmosuchian *Lobalopex* has a large prootic that forms the lateral wall of much of the posterior portion of the braincase (Sidor, Hopson & Keyser, 2004). The hypophyseal fossa is laterally surrounded by the prootic, and the prootics meet posteriorly (Sidor, Hopson & Keyser, 2004). Boonstra (1968) demonstrated for various dinocephalians that the prootics meet at the midline via a process that forms the dorsal portion of the dorsum sella, where the ventral portion is formed by the basisphenoid. This is also the condition observed in the gorgonopsian specimens here described. However, there is no separation of the parabasisphenoid complex into different ossifications in dinocephalians (Boonstra, 1968). A separate ossification between the basioccipital and parasphenoid-basipresphenoid has been demonstrated for the dicynodonts *Niassodon* (Castanhinha et al., 2013), *Lystrosaurus* (Cluver, 1971), and it is also present in GPIT/RE/9275. Dicynodonts do not have the two prootics meeting at the skull midline (Camp & Welles, 1956; Boonstra, 1968; Surkov & Benton, 2004; Castanhinha et al., 2013; Cluver, 1971). The prootics crista alaris, contacting the supraoccipital posteriorly and the pila antotica, raise anterodorsally from the prootics base. This condition is seemingly a reversal from the more general condition in Synapsida. It is apparent that the basicranium was under substantial morphological change among therapsids, despite the limited knowledge on more basal synapsids. Therocephalians have been described as possessing a midline contact of the prootics that forms the dorsum sella (Boonstra, 1968; Boonstra, 1971; van den Heever, 1994). From our observations for GPIT/RE/7139, the sella turcica in therocephalians is anteroposteriorly elongated and the prootics contact slightly in the midline, and the parabasisphenoid is a single fused element (Figs. 19C, 19D). Further observations are required to assess the ontogenetic development of the parabasisphenoid in osteologically immature specimens. The sellar region in basal cynodonts has striking resemblance with that of therocephalians, with an elongated and shallow sella turcica, however, the dorsum sella is shallow and formed by the basisphenoid (e.g., BP1-5973 see Video S1 Rigney, 1938; Fourie, 1974; Kemp, 1969). On the other hand, the prootics are well separated from one another in the sagittal plane (BP1-5973 see Video S1, Rigney, 1938; Fourie, 1974; Kemp, 1969), resembling the anomodont condition. In a rare example, the synchrotron scans of *Thrinaxodon liorhinus* (BP/1/7199) and *Galesaurus* (BP1-5973 see Video S1) show the separation between the dermal parasphenoid and the endochondral basisphenoid. A thin sheet of the parasphenoid envelops the posterior portion of the basisphenoid trabecular bone. In these specimens, the basisphenoid and the basioccipital are conspicuously separated by a gap (“unossified zone” of Olson (1944) and Fourie (1974)). A similar gap is filled with basal plate cartilage in other tetrapods (Paluh & Sheil, 2013). The basal cynodont basicranium closely resembles the mammalian condition. In the basal mammaliaform *Morganucodon* (Kermack, Mussett & Rigney, 1981), or in the more derived *Triconodon* (Kermack, 1963), the prootics are separated by a broad basisphenoid. Similarly, in mammals the petrosals/periotic (prootic + opisthotic) form a rather lateral position in the braincase (Novacek, 1993).

An important implication of the sellar region reorganization is the modification of the abducens nerve path as well as the extraocular musculature, namely the retractor bulbi group. In reptiles, the retractor bulbi muscles attach on the clinoid processes of the basisphenoid dorsum sella (Säve-Söderbergh, 1946). In mammals, on the other hand, the retractor bulbi muscles insert on the orbital exposure of the basisphenoid (e.g., Porter et al., 1995). If we use the reptilian configuration as the plesiomorphic condition, it follows that either the structural dorsum sella formed by the prootics medial process began to serve as the attachment area of the retractor bulbi or these muscles, or that the retractor bulbi inserted on a more lateral aspect of the saddle-shaped dorsal buttresses of the parasphenoid-basipresphenoid tentatively homologized with the processus clinoideus (see description). However, the topology of these structures does not allow us to rule out they may be the rostrum basisphenoidale (Paluh & Sheil, 2013). The hypothesis of attachment site adjustment from the basisphenoid to the prootics medial projection does not seem to be convincing because the retractor bulbi musculature has highly conservative origin loci across tetrapods (Walls, 1942). On the other hand, we favor the hypothesis of a small lateral readjustment of the retractor bulbi musculature towards the saddle shaped buttresses on the parasphenoid-basipresphenoid complex because it is a more parsimonious explanation. Otherwise, the origin of the retractor bulbi muscles would have to change from the basisphenoid to the prootics in non-anomodont therapsids, and then subsequently change back to the basisphenoid in mammals.

Although carotid circulation has been studied in detail for cynodonts and mammaliaforms (Rougier & Wible, 2006; Müller, Sterli & Anquetin, 2011), little is known for more basal synapsids. Theriodontia share a unique condition among synapsids in having the cerebral branch of the internal carotid exiting as a single opening on the anteriormost portion of the sella turcica. This can be attested for GPIT/7124 and GPIT/RE/7199 for gorgonopsians, GPIT/RE/7139 and van den Heever (1994) for therocephalians, BP/1/7199 and Fourie (1974) for cynodonts. The condition in cynodonts and therocephalians is slightly different from gorgonopsians. In cynodonts and therocephalians the two cerebral branches perforate the parabasisphenoid ventrally and then subsequently coalescing at about halfway towards the dorsal side of the basisphenoid, whereas in gorgonopsians the two cerebral branches perforate the parabasisphenoid laterally follow a horizontal path towards the median part of the skull and then coalesce at the sagittal plane. However, the parabasisphenoid region in gorgonopsians is much deeper. Burnetiamorphs, dinocephalians, anomodont, but also mammaliaforms have two perforations on the sella turcica for the cerebral branches of the internal carotids, thus, exiting separately (e.g., Boonstra, 1968; Crompton & Museum, 1958; Kermack, 1963; Kermack, Mussett & Rigney, 1981).

### The enigmatic posterior ossification of the basisphenoid

**Figure 20:**
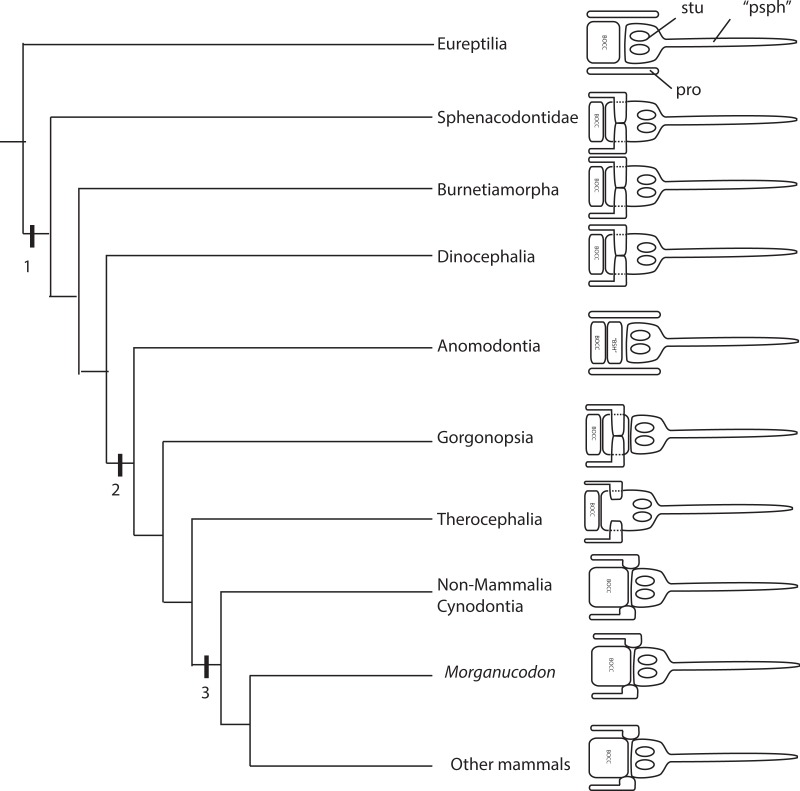
Major anatomical and developmental transformations of the parasphenoid, basisphenoid, prootic, basioccipital complex in synapsids: 1—Synapsida: Morphology: formation of the medial prootic process, prootics meet medially; Development: invasion of the otic capsule cartilage onto the basal plate region. 2—Theriodontia: Morphology: reorganization of the prootic and parabasisphenoid complex; basipostsphenoid becomes a separate ossification; Development: shift of the neural crest—mesoderm boundary (or prechordal-chordal skull boundary). 3—Cynodontia: Morphology: petrosal (opishtotic + prootic) contacts parabasisphenoid complex Development: possible supression of the mesoderm-derived posterior portion of the basisphenoid due to induction of the otic capsule cartilage. Abbreviations: bocc, basioccipital; “bsh” basisphenoid but here basipostsphenoid; pro, prootic; “psph”, parasphenoid but here parasphenoid + basi-presphenoid; stu, sella turcica.

The parasphenoid-basisphenoid is a complex element in most vertebrates, formed from a number of different ossifications of chondrocranial and dermatocranial origins. The complexity of this region leads to nomenclatural problems arising from both homologous bones being named differently in the major tetrapod groups (i.e., reptiles, birds, mammals) and evolutionary shifts in developmental programs, yielding identification of homologous elements difficult (Fig. 20).

Fate mapping experiments show a fundamental reorganization of the braincase bones among vertebrates (Couly, Coltey & Le Douarin, 1993; Jiang et al., 2002; Noden & Trainor, 2005; Kague et al., 2012; Piekarski, Gross & Hanken, 2014). For instance, the parasphenoid can be confused with the vomer (Atkins & Franz-Odendaal, 2015), or the basipresphenoid in the chick does not seem to be homologous with the presphenoid bone in the mouse (McBratney-Owen et al., 2008). It is thus, crucial to understand the therapsid origins of the parasphenoid and basisphenoid to shed light on the mammalian evolution.

The parasphenoid and the basisphenoid are typically described separately in the gorgonopsian literature (e.g., Sigogneau, 1970; Sigogneau-Russell, 1989; Kammerer et al., 2015; Kammerer, 2016). However, co-ossification of the two bones and the fact that typically only the ventral view of these bones is visible, the parasphenoid refers exclusively to the cultriform process (or parasphenoid rostrum), and the basisphenoid to the basal tubera, thus rendering difficulties to understand the exact delimitation of each bone. Notably, the structures that compose the dorsal view of these bones have not been described.

In the synapsid outgroup, the typical reptilian braincase configuration comprises the basisphenoid which is typically fused with the cultriform process anteriorly and the lateral wings of the parasphenoid ventrally (Gardner et al., 2010; Sobral, Sues & Müller, 2015), the degree of fusion of these elements leads various authors to describe this element as the parabasisphenoid. It is consensual though that the basisphenoid is perforated by the internal carotids dorsally and excavated by the sella turcica, and bearing the dorsum sella posteriorly (Rieppel, 1993). Lateral to the basisphenoid lay the prootics, and it is often posteriorly fused with the basioccipital.

Importantly, the sella turcica is a highly conservative structure laying universally in vertebrates on the basisphenoid (Hanken & Hall, 1993). However, surprisingly, in his extensive monograph on gorgonopsian anatomy Kemp (1969) described the sella turcica and the internal carotid foramina as part of the parasphenoid. Indeed, the median ridge of the sella turcica are described just posterior to the parasphenoid cultriform process posterior border (Kemp, 1969, see Fig. 7 for *Leontocephalus* and p. 64 for *Arctognathus*). Furthermore, he notes that the prootics have medial processes that meet in the sagittal plane of the skull, thus excluding the posterior part of the parasphenoid-basisphenoid complex to form the dorsum sella (similar to “pelycosaurs” Romer & Price, 1940). A similar anteriorly-shifted sella turcica is present in the gorgonopsian outgroup: the dicynodonts (Cluver, 1971; Castanhinha et al., 2013). This unique configuration of the braincase has remained unquestioned. However, if we accept that the sella turcica sits on the parasphenoid, such braincase arrangement represents a dramatically different reorganization of the skull, because highly conservative structures such as the sella turcica and dorsum sella modified their typical loci.

The separate, intermediate ossification between “Kemp’s parasphenoid” and the basioccipital in the osteologically immature (see Araújo et al., 2015) skull of GPIT/RE/7124 provides significant insights into the homology and ossification sequence of these structures within synapsids. The dermal bone parasphenoid fuses early in ontogeny with the anterior ossification center of the basisphenoid which has the neural crest-derived trabeculae as the cartilaginous precursor. The processus clinoideus and the sella turcica are thus formed on the basisphenoid. The mesoderm-derived trabeculae cartilages are the precursors to the posterior ossification center of the basisphenoid, which is a distinct ossification in the immature GPIT/RE/7124 specimen.

Unexpectedly, the prootics, which originate from a different cartilaginous precursor (the otic capsule), meet at the skull midline posterior to the hypophysis and the trabeculae cartilage region (Fig. 20). The prootics do not floor the braincase in the typical reptilian configuration (Rieppel, 1993), but occupy a more lateral position, the posterior part of the basisphenoid flooring the braincase. In the specimen described here, the medial processes meet at the midline at the level of the posterior ossification center of the basisphenoid, here called the basipostsphenoid. This explains why Kemp (1969) labeled these processes the dorsum sella, due to their topological position relative to the sella turcica. Thus, Kemp’s nomenclature is strictly a structural/positional term and not homologous to the dorsum sella which has its chondrocranial origin as the acrochordal cartilage in various tetrapod groups (Sheil, 2005; Säve-Söderbergh, 1946; McBratney-Owen et al., 2008; Jollie, 1957; Crompton, 1953).

Notwithstanding the developmental origins and nomenclatural aspects of the part of the bone bounding the posterior part of the sella turcica, this configuration in the specimens described here suggests a peculiar developmental pattern affecting the otic capsule and basal plate cartilages and was widespread in the synapsid lineage. Possibly,the medial development of the otic capsule-derivative, the prootics in this case, induced developmental suppression of the mesoderm-derived posterior ossification center of the basisphenoid.

### The gorgonopsian brain in the context of synapsid brain evolution

We here provide the first digital endocast of a gorgonopsian brain (Fig. 16). In recent publications, both anomodont and therocephalian endocasts provided insights on non-cynodont neotherapsids brain morphology (Sigurdsen et al., 2012; Castanhinha et al., 2013). Various publications provided also pertinent information on the endocranial cavities of cynodonts (Quiroga, 1980; Quiroga, 1984; Rodrigues, Ruf & Schultz, 2013). However, the critical phases of the synapsid brain evolution happened later in two pulses exemplified by the endocasts of *Morganucodon* and *Hadrocodium* (Rowe, Macrini & Luo, 2011). Neither anomodonts (Castanhinha et al., 2013), nor gorgonopsians (this paper), nor therocephalians (Sigurdsen et al., 2012) show any signs of the expansion of the neocortex and elevated encephalization coefficients to mammalian levels. Indeed, our findings support that pre-mammaliaform brain morphology and volume remained conservative, even among derived cynodonts (Ulinski, 1986; Rodrigues, Ruf & Schultz, 2013; Rowe, Macrini & Luo, 2011). Indeed, the enlarged hindbrain relative to the forebrain, the large epiphyseal nerve, the large hypophysis, and the elongate shape of the brain endocast are conservative among non-mammaliaform neotherapsids, sharing a more general aspect with a reptilian-grade brain. However, some derived features visible in basal cynodonts are not present in the gorgonopsian representation here provided, namely the anterior colliculi (Quiroga, 1980).

Kemp (1969) attempted to reconstruct the brain endocast from a variety of different specimens from different species, rendering difficult direct comparisons with the endocast described here. However, some differences from our reconstruction are conspicuous, namely in the hypophysis and epiphyseal nerve. The brain endocast of GPIT/RE/7124 differs from that of Kemp (1969) as he reconstructed a highly-elongated, posteriorly-oriented hypophysis. The GPIT/RE/7124 hypophysis endocast is vertically-oriented and a rather short and stout depression in the basipresphenoid.

There is no evidence of a parapineal organ anterior to the pineal organ, as suggested by Kemp (1969). The epiphyseal nerve in GPIT/RE/7124 exits through a single oval opening bounded by the parietals. Additionally, Kemp (1969) estimates the exit of the optic nerve (cnII) near the junction between the forebrain and midbrain. However, as he noted for his specimens, there is also no evidence in GPIT/RE/7124 for any osseous enclosure of the optic nerve.

The floccular complex lobes are proportionally large compared with the estimated brain endocast volume in the gorgonopsian taxon studied here. However, a recent study showed that ecology or function does not correlate with floccular size (Ferreira-Cardoso et al., in press). Although morphologically well-delimited, the flocculus is rather a functionally-integrated structure with the rest of the cerebellum, notably for gaze stabilization and vestibule-ocular reflex (Ferreira-Cardoso et al., in press).

**Figure 21:**
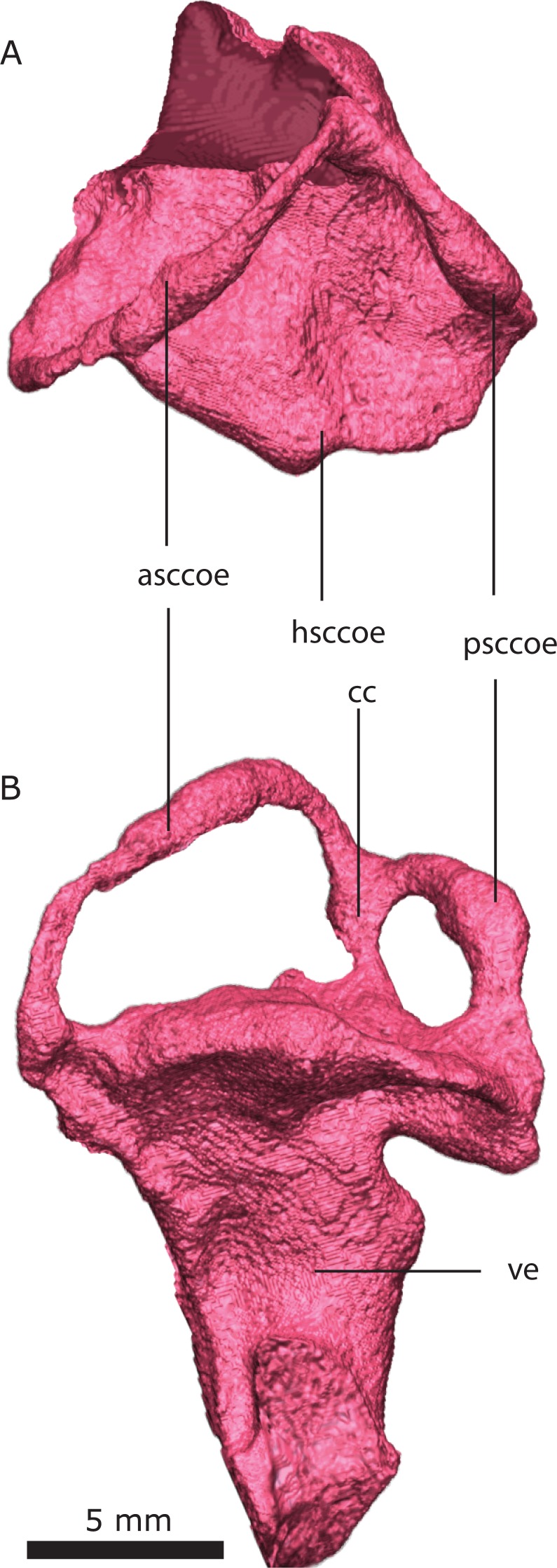
Left osseous in dorsal (A) and lateral (B) views. Abbreviations: asccoe, anterior semicircularcanal osseous enclusure; cc, crus communis; hsccoe, horizontal semicircular canal osseous enclusure; psccoe, posterior semicircular canal osseous enclusure; ve, vestibule.

### Osseous labyrinth

Olson (1938) and Olson (1944) was the first to study the inner ear of gorgonopsians. The anatomy of the model of the membranous labyrinth presented by Olson (1938) is substantially different from the endocast presented here (Fig. 21). For instance, the extensive development of the ampullae, the anterior and posterior semicircular canals being subequal in size, there is a high degree of torsion of the horizontal semicircular canal, and the crus communis is subtriangular in shape tapering dorsally (Olson, 1938, Fig. 2). Furthermore, most of the features described on the membranous labyrinth (e.g., utriculus, sacculus) cannot be discerned from the osseous enclosure of the labyrinth. However, a second attempt was performed by Sigogneau (1974) also using serial grinding techniques to reconstruct the osseous labyrinth of *Gorgonops* (BP/1/277). Although the model resulting from the grinding techniques does not seem to be in total accordance with ours (e.g., development of the ampullae, location and development of the osseous enclosure of the utriculus and sacculus, “doubling” of the anterior semicircular canal, the osseous enclosure of the labyrinth done by the opisthotic exclusively), various observations done by Sigogneau (1974) and Sigogneau-Russell (1989) are in agreement with our findings (Fig. 21). Notably, the oblique orientation of the entire vestibular organ with respect to the cranial axis, the absence of ossification of the horizontal semicircular canal, the partially open canal of the anterior semicircular canal, poor development of the osseous enclosure of the ampullae, and the longer anterior semicircular canal relative to the posterior (Fig. 21, Sigogneau, 1974; Sigogneau-Russell, 1989).

### Head posture in gorgonopsians

**Figure 22:**
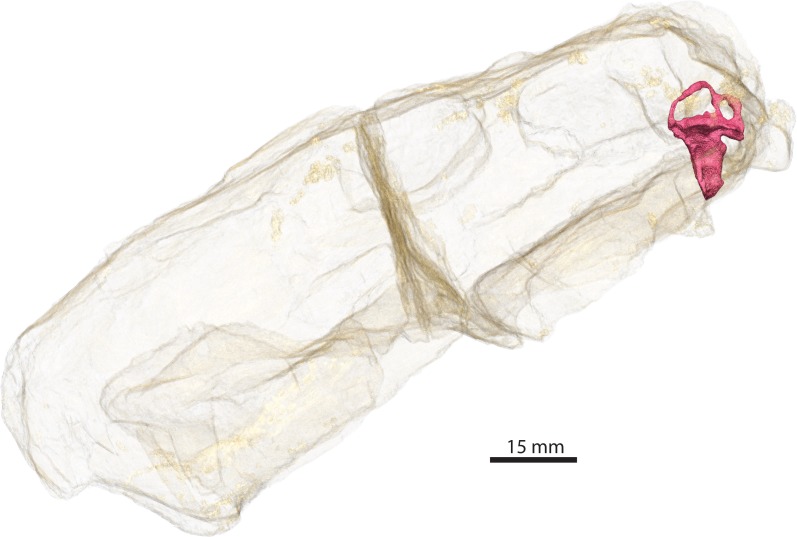
Head posture of GPIT/RE/7124 if the horizontal semicircular canal is aligned with earth plane. Note the anteriorly tilted neutral head posture if the horizontal SC is aligned to the Earth’s surface plane.

The orientation of the horizontal semicircular canal has been used to estimate the habitual alert head posture (Lebedkin, 1924; Duijm, 1951; Rogers, 1998; Evans, 2006). Although questions have been raised concerning this assumption (Marugán-Lobón, Chiappe & Farke, 2013), even in the extreme case of the sauropod *Nigerasaurus,* the head is still tilted forward after the Procrustes methods proposed by the authors had been applied. Indeed, most authors agree that the alert posture requires a leveled horizontal semicircular canal or slightly elevated in the front in about 5–10° (Lebedkin, 1924; Duijm, 1951; Erichsen et al., 1989; Witmer et al., 2003). If the horizontal semicircular canal is aligned relative to the earth’s surface plane, this implies that the head of GPIT/RE/7124 is tilted by 41° (Fig. 22). This ventrally-tilted head posture has been related to binocular vision, allowing for a greater overlap of the visual fields (Witmer et al., 2003), consistent with the predatory habits of gorgonopsians.

### The closed osseous enclosure of the horizontal semicircular canal

The horizontal semicircular canal is discoid instead of the typical toroid shape (Fig. 21). This is consistent in both sides of the skull, and was also reported in *Gorgonops* by Sigogneau (1974), and suggests we can rule out skull deformation and distortion to explain this unique anatomy, unique amongst reptiles and mammals.

The functional implications of this distinctive morphology are difficult to understand. The membranous labyrinth typically runs close to the outer wall of the bony labyrinth; therefore, it seems unlikely that the membranous labyrinth occupied a deeper position.

The horizontal semicircular canal lays in a deep excavation on the dorsal surface of the opisthotic (Fig. 9) and there is a similarly deep excavation in the dorsal surface of the supraoccipital (Fig. 10). Therefore, the horizontal semicircular canal is wedged in between these two bones. Arguably, spatial or possibly developmental constraints (or both) prevent the typical toroidal configuration of the horizontal semicircular canal.

##  Supplemental Information

10.7717/peerj.3119/supp-1 Video S1 Galesaurus basicranium BP1-5973

## Institutional Acronyms

BPI: Evolutionary Studies Institute (formerly Bernard Price Institute) University of Witwatersrand, Johannesburg, South Africa
GPIT: Institut und Museum für Geologie und Paläontologie, Eberhard Karls Universität, Tübingen, Germany
AMNH: American Museum of Natural History, New York, USA
RC: Rubidge Collection, Wellwood, South Africa
NHMUK: Natural History Museum, London, United Kingdom
UMZC: University Museum of Zoology, Cambridge, United Kingdom

## Additional Information and Declarations

### Data Availability

The following information was supplied regarding data availability: MorphoBank Project 2503
https://morphobank.org/index.php/Projects/ProjectOverview/project_id/2503.
